# A genetic screen for modifiers of cohesin clustering identifies regulators of genome folding

**DOI:** 10.1126/sciadv.adx5130

**Published:** 2026-01-30

**Authors:** Wonho Kim, Daniel S. Park, Son C. Nguyen, Rachel Yang, Eric F. Joyce, Rajan Jain

**Affiliations:** ^1^ Epigenetics Institute, University of Pennsylvania, Philadelphia, PA, USA.; ^2^ Department of Medicine, Department of Cell and Developmental Biology, Institute of Regenerative Medicine, Penn Cardiovascular Institute, Perelman School of Medicine, University of Pennsylvania, Philadelphia, PA, USA.; ^3^ Department of Genetics, Integrative Program for Biological and Genome Sciences, University of North Carolina, Chapel Hill, NC, USA.; ^4^ Department of Genetics, Perelman School of Medicine, University of Pennsylvania, Philadelphia, PA, USA.

## Abstract

The cohesin complex orchestrates 3D genome architecture through multiple steps including loading onto chromatin, DNA loop extrusion, stalling of extrusion, and unloading off chromatin. However, the upstream regulatory factors modulating these steps remain largely unexplored. Previous studies suggest that cohesin clustering correlates with its chromatin residence time and loop extrusion activity. Here, we developed, optimized, and performed an imaging-based genetic screen leveraging modulation of cohesin clustering to identify cohesin regulators. Using a sensitized background in which the cohesin unloader WAPL is partially degraded, we screened the druggable genome for effects on cohesin clustering. Through multiple rounds of screening and experimentation, we identified 7 enhancers and 10 suppressors of cohesin clustering. Several factors control genome folding at multiple loci and cohesin loading. Notably, our screen identified factors in mitochondrial function and epigenetic silencing, implicating these processes in the regulation of cohesin activity. This study offers a valuable resource identifying cohesin regulators and provides insights into upstream mechanisms governing genome folding.

## Teaser

A large-scale imaging study identified factors involved in protein modification and gene regulation as genome folding regulators.

## INTRODUCTION

The genome must be intricately folded to fit within the nucleus and emerging evidence shows genome folding is highly regulated (*1*, *2*). Proper genome folding is required for normal gene expression and DNA repair (*3*–*5*); disruptions in genome folding are linked to various diseases including cancer and developmental disorders (*5*–*7*). Therefore, elucidating mechanisms governing genome folding is critical for understanding essential regulatory processes for cellular health.

**Fig. 1.**
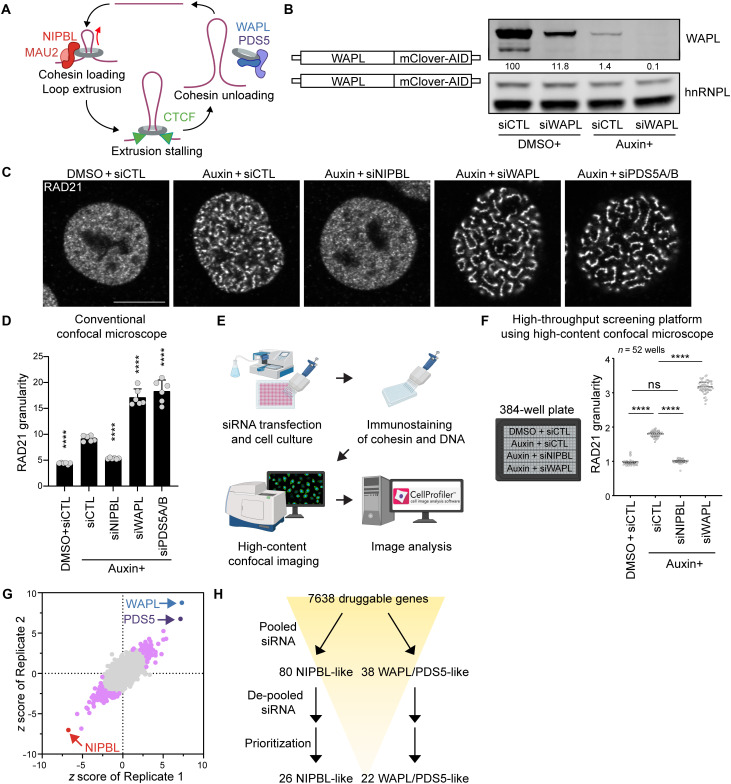
Design and primary screen to identify factors controlling cohesin clustering. (**A**) Schematic model of cohesin-mediated genome folding, illustrating roles of NIPBL, MAU2, WAPL, PDS5, and CTCF in cohesin loading, loop extrusion, unloading, and extrusion stalling. Created in BioRender. W. Kim (2025) https://BioRender.com/y254inl. (**B**) Generation of HCT116:WAPL-AID cell line (left). WAPL immunoblots of HCT116:WAPL-AID cells treated with DMSO or auxin (1 μM, 72 hours) and transfected with nontargeting (CTL) siRNA or WAPL-targeting siRNA (right). hnRNPL was used as a loading control. Relative normalized WAPL band intensity (%) is shown. (**C**) RAD21 immunostaining of HCT116:WAPL-AID cells treated with auxin (1 μM, 72 hours) and transfected with siRNAs targeting NIPBL, WAPL, or PDS5A/B. Representative images obtained from conventional confocal microscopy. Scale bar, 10 μm. (**D**) Quantification of RAD21 granularity from (C). ****P < 0.0001 [analysis of variance (ANOVA) with Dunnett’s test compared to nontargeting siRNA with auxin treatment]. *n* = 6. Error bars denote mean + SD. (**E**) Schema of high-throughput screening platform. Created in BioRender. W. Kim (2025) https://BioRender.com/nlvhdp5. (**F**) RAD21 immunostaining and granularity analysis using high-throughput pipeline in a 384-well plate. HCT116:WAPL-AID cells were treated with DMSO or auxin (1 μM, 72 hours) and transfected with nontargeting siRNA or siRNAs targeting NIPBL or WAPL (52 wells per condition). Quantification of RAD21 signal granularity is shown on the right. RAD21 granularity was measured per nucleus, and the median per well was plotted. The median (thick dotted line) and interquartile range (thin dotted lines) are indicated. ****P < 0.0001; ns, nonsignificant (ANOVA with Tukey’s test). (**G**) Pooled siRNA primary screen results. *z* scores for RAD21 granularity from two replicates are shown (Pearson correlation of 0.80). Hits prioritized for further studies are highlighted (80 NIPBL-like and 38 WAPL/PDS5-like, magenta). (**H**) Primary screening workflow summarizing pooled siRNA screen, de-pooled siRNA validation, and hit prioritization.

Genomic proximity-ligation mapping methods like Hi-C identified the cohesin complex as a key driver of genome folding (*8*, *9*). Moreover, single-molecule imaging showed that cohesin is a molecular motor directly driving DNA loop extrusion in vitro (*10*, *11*). Cohesin folds the genome in a stepwise manner and is regulated by various factors (Fig. 1A). NIPBL and MAU2 promote both cohesin loading onto chromatin and cohesin loop extrusion activity by stimulating core cohesin ring adenosine triphosphatase activity (*10*, *12*–*16*). Loss of NIPBL leads to reduced cohesin levels on chromatin and reduced numbers of chromatin loops (*14*). WAPL facilitates cohesin unloading, and WAPL depletion leads to prolonged cohesin residence time on chromatin and an increase in both loop number and length (*9*, *17*, *18*). PDS5 functions with WAPL to unload cohesin (*19*, *20*), and simultaneous depletion of both PDS5 paralogs (PDS5A and PDS5B) extends the residence time of cohesin on chromatin and increases loop length, similar to WAPL loss (*9*). In contrast to WAPL depletion, PDS5 depletion reduces the number of loops, suggesting additional functions for PDS5 beyond WAPL cooperation (*9*, *21*, *22*). Last, CTCF (CCCTC-binding factor) binds the base of loops, often coinciding with topologically associated domain (TADs) boundaries (*23*). CTCF depletion results in weaker CTCF-anchored loops and TAD boundaries, suggesting CTCF functions to stall cohesin-mediated loop extrusion and stabilize chromatin loops (*9*, *24*, *25*).

Cohesin loop extrusion activity in vivo is supported by observations of cohesin clustering into axial structures termed vermicelli (“little worms”) in WAPL knockout (KO) cells (*26*). The absence of WAPL prevents cohesin unloading, resulting in continuous translocation along chromatin and clustering of cohesin molecules. Accordingly, depletion of NIPBL and MAU2 suppresses cohesin clustering in WAPL KO cells (*17*). Similarly, depletion of PDS5A and PDS5B also results in cohesin clustering (*9*).

While mechanisms underlying the activity of cohesin and related factors have been extensively studied during these steps, the upstream molecular mechanisms regulating cohesin function remain elusive. A detailed understanding of these mechanisms is crucial for understanding if and how the various steps of the cohesin life cycle can be regulated, and more broadly, how cohesin-mediated genome structure controls cellular function. Recent studies identified BRD4 (bromodomain containing 4) (*27*), GSK3A (glycogen synthase kinase 3 alpha) (*28*), and YY1 (YY1 transcription factor) (*29*–*31*) as regulators of genome folding. Genetic screens offer a powerful approach to extend these studies and identify additional regulators of cohesin. Hi-C and similar techniques are cost-prohibitive and cumbersome for high-throughput applications. Therefore, we established and conducted a high-throughput imaging-based genetic screen assaying cohesin clustering. Our robust imaging platform and analysis pipeline enabled efficient screening of more than 7600 druggable genes in a high-throughput manner. Through multiple rounds of screening, we identified multiple potential NIPBL-, WAPL- and PDS5-like factors. We demonstrate several of these factors affect genome folding across multiple genomic loci and alter levels of chromatin-bound cohesin. Last, we provide evidence showing some factors may directly regulate cohesin and/or its associated factors. Together, this genetic screen provides a valuable resource for identifying potential regulators of cohesin and offers critical insights into the diversity of pathways that govern genome folding.

## RESULTS

### Cohesin clustering upon partial WAPL degradation is modulated by known cohesin regulators

WAPL KO cells exhibit a wormlike cohesin clustering phenotype (vermicelli) dependent on NIPBL and MAU2 (*17*). To investigate this genetic interaction, we degraded WAPL in an auxin-inducible degron (AID) cell line (HCT116:WAPL-AID) where the AID tag is fused to endogenous WAPL (Fig. 1B) (*28*). WAPL was mostly, but not completely, reduced after 72 hours of 5-phenyl-indole-3-acetic acid (5-Ph-IAA; modified auxin, hereafter referred to as auxin) administration (Fig. 1B). Near-complete WAPL depletion was achieved with 72 hours of simultaneous auxin treatment and WAPL-targeting siRNA transfection as determined by immunoblotting. WAPL-depleted cells continued to proliferate, allowing for sufficient cells for screening, though the growth rate was 30% slower after 3 days of auxin (fig. S1A).

We observed cohesin clustering as early as 5 hours after auxin administration, which increased over time (fig. S1B). We quantified clustering by measuring granularity of RAD21 immunofluorescent signal, a measure for pixel intensity relative to the local background (see Materials and Methods) using a conventional confocal microscope. We depleted factors involved in cohesin processivity via siRNA to determine whether cohesin clustering could be modulated by altering cohesin loading, loop extrusion activity, or unloading. Simultaneous auxin administration with NIPBL knockdown (72 hours) nearly completely suppressed clustering, while simultaneous auxin administration with residual WAPL or PDS5A/B knockdown exacerbated cohesin clustering (Fig. 1, C and D, and fig. S1C). These results indicate cohesin clustering induced by partial WAPL loss can be modulated by factors regulating cohesin loading, loop extrusion activity, and unloading.

### A high-throughput genetic screen identifies factors modulating cohesin clustering

On the basis of the genetic interaction we observed, we hypothesized regulators of cohesin activity could be identified by screening for modulators of cohesin clustering in cells with partial WAPL degradation. To perform a systematic screen, we established a high-throughput pipeline with cell culture in 384-well plates followed by RAD21 immunostaining, automated imaging with a high-content confocal microscope, and image analysis to quantify cohesin clustering in individual nuclei (Fig. 1E and fig. S1D). To determine the reproducibility of clustering across dozens of replicates, we seeded HCT116:WAPL-AID cells in a 384-well plate and transfected with siRNA against NIPBL, WAPL, or nontargeting control, in the presence or absence of auxin (*n* = 52 wells each condition; Fig. 1F). After 72 hours, we performed RAD21 immunofluorescence, imaged, and analyzed RAD21 granularity across >200 nuclei per well to compare cohesin clustering across wells. Partial WAPL depletion via auxin significantly increased clustering compared to vehicle controls (Fig. 1F). NIPBL knockdown in auxin-treated cells suppressed clustering to levels comparable to the nontargeted vehicle controls. In contrast, residual WAPL knockdown via siRNA in auxin-treated cells further enhanced clustering compared to auxin treatment alone. RAD21 granularity scores were highly consistent across wells within the same condition (Fig. 1F), indicating that the platform could robustly identify suppressors and enhancers of cohesin clustering in a 384-well format.

Having established conditions to measure cohesin clustering in an automated fashion, we conducted a high-throughput siRNA screen in duplicate targeting 7638 druggable genes (fig. S1E and table S1). We first used multiplexed siRNAs combining four siRNAs per gene to maximize knockdown efficiency. siRNAs targeting NIPBL, WAPL, and PDS5A/B were manually added. Cohesin clustering was visually distinguishable across a wide range of *z* scores, allowing for clear identification of potential hits (fig. S2A). In total, we screened 8960 wells, including putative hits and controls, of which 87.5% were retained for analysis. We further confirmed that there was no positional bias in *z* scores across the 384-well plate (fig. S2B), and observed a strong correlation between biological duplicates (Pearson correlation, 0.80; Fig. 1G).

NIPBL was the strongest suppressor of cohesin clustering, while WAPL and PDS5A/B were the strongest enhancers (Fig. 1G). Auxin-mediated degradation requires *Oryza sativa* TIR1 (Transport Inhibitor Response 1) to form an SCF (Skp1-Cul1-F-box) E3 ligase complex with endogenous proteasomal machinery (*32*). SKP1, RBX1, and CUL1, respectively, ranked as the first, seventh, and eight strongest suppressors of cohesin clustering, suggesting WAPL protein was not degraded by auxin treatment in these knockdowns (fig. S2C). Therefore, we excluded genes related to the auxin degron and proteasome machinery from further analysis. In total, we prioritized 80 suppressors (NIPBL-like) and 38 enhancers (WAPL/PDS5-like), representing 118 genes in total for further validation and experimentation (1.5% of the screened genes; Fig. 1H; see Materials and Methods).

Next, we studied the 118 putative hits by re-screening with individual siRNAs (4/gene) in biological triplicate. Granularity was consistent across replicates for both suppressors and enhancers (Pearson correlation >0.90 for all comparisons; fig. S2, D and E). We expected knockdown of bona fide suppressors and enhancers to result in an increase or decrease in granularity beyond the nontargeting siRNA range. Ninety-eight percent of nontargeting siRNA replicates fell within ±7% of the median, whereas approximately 50% of siRNAs targeting candidate genes were outside this range (fig. S2F and table S1). Given the distribution of data skewing toward WAPL/PDS5-like hits, we further prioritized the hits to study as follows: For NIPBL-like hits, the most effective siRNA decreased clustering by >20% and the second best by >10%; for WAPL/PDS5-like hits, the most effective siRNA increased clustering by >30% and the second best by >10%. On the basis of these thresholds, we identified 26 NIPBL-like and 22 WAPL/PDS5-like hits from our primary screen efforts (Fig. 1H and table S1). Supporting the robustness of these hits, 22 of 26 NIPBL-like hits and 20 of 22 WAPL/PDS5-like hits showed consistent clustering phenotypes supported by more than two of the four siRNAs tested, albeit occasionally with a smaller effect size (fig. S2, G and H, and table S1).

### Cohesin clustering in secondary screens using orthogonal depletion of WAPL

**Fig. 2.**
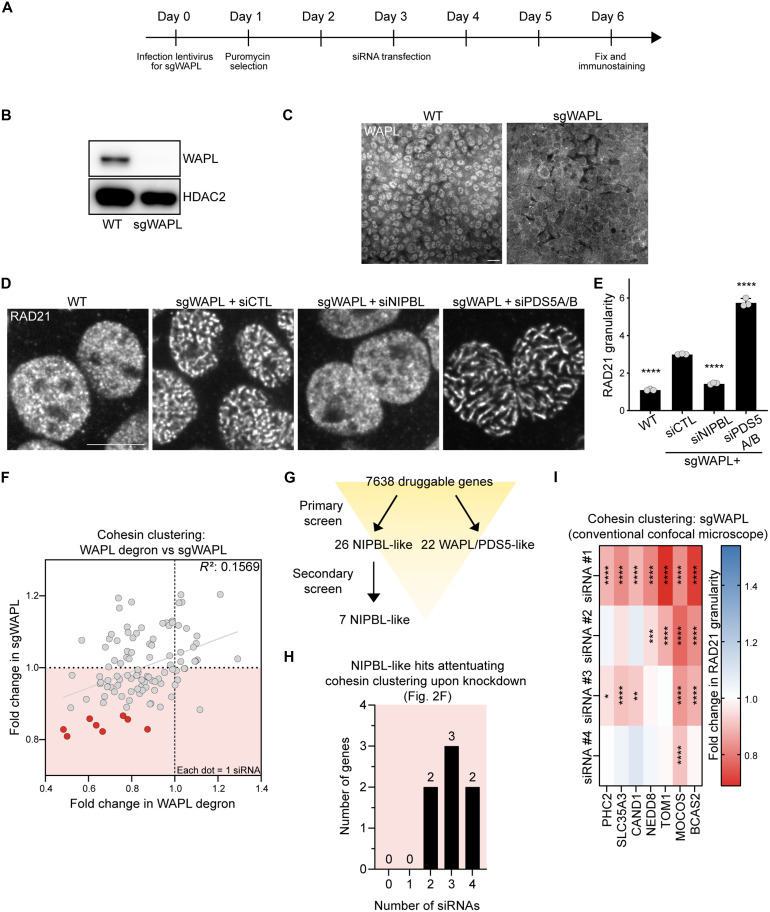
Secondary screen of NIPBL-like hits. (**A**) Experimental timeline for secondary screen using CRISPR-generated WAPL KO cells. (**B**) Immunoblots of WAPL from HCT116 cells expressing sgRNAs targeting WAPL. HDAC2 was used as a loading control. (**C**) WAPL immunostaining of HCT116 cells expressing sgRNAs targeting WAPL. Scale bar, 10 μm. (**D**) RAD21 immunostaining of wild-type (WT) or CRISPR WAPL KO cells transfected with nontargeting (CTL) siRNA or siRNA targeting NIPBL or PDS5A/B. Scale bar, 10 μm. (**E**) Quantification of RAD21 granularity from (D). ****P < 0.0001, as determined by ANOVA followed by Dunnett’s test to compare conditions to the nontargeting siRNA with sgRNA against WAPL. *n* = 3. Error bars denote mean + SD. (**F**) Scatter plot showing relationship between fold changes in RAD21 granularity from the primary screen using WAPL degron and the secondary screen using WAPL KO cells. Data points represent individual siRNAs targeting one of 26 NIPBL-like hits, with four siRNAs per hit. siRNAs passing a stringent threshold (fold change <0.87 in WAPL KO and <0.9 in WAPL degron) are shown as red circles. Simple linear regression line is included (*P* < 0.0001), and *R*^2^ value is indicated. (**G**) Screening workflow summarizing primary and secondary screens for identifying seven NIPBL-like hits. (**H**) Numbers of genes with zero to four individual siRNAs showing decreased RAD21 granularity in the secondary screen for seven NIPBL-like hits [siRNAs in the light-red area of (F)]. (**I**) Heatmap of RAD21 granularity fold change for four individual siRNAs (columns) targeting seven NIPBL-like hits (rows) in WAPL KO cells. Data were obtained using a conventional confocal microscope. Color intensity indicates the magnitude of fold change. *P < 0.05, **P < 0.01, ***P < 0.001, and ****P < 0.0001, as determined by ANOVA followed by Dunnett’s test to compare conditions to nontargeting siRNA.

We used CRISPR-Cas9 to genetically KO WAPL in HCT116 cells (hereafter WAPL KO cells) as an alternative method of WAPL depletion to identify genuine regulators of cohesin clustering. Three days after introducing two gRNAs targeting WAPL, we transfected individual siRNAs, and assayed cohesin clustering as above (Fig. 2A). We confirmed efficient WAPL deletion across the vast majority of cells by immunoblotting and immunostaining (Fig. 2, B and C). Similar to auxin-mediated WAPL degradation, WAPL KO induced cohesin clustering (Fig. 2, D and E). Clustering was significantly suppressed by NIPBL knockdown and enhanced by PDS5A/B depletion in WAPL KO cells (Fig. 2, D and E).

**Fig. 3.**
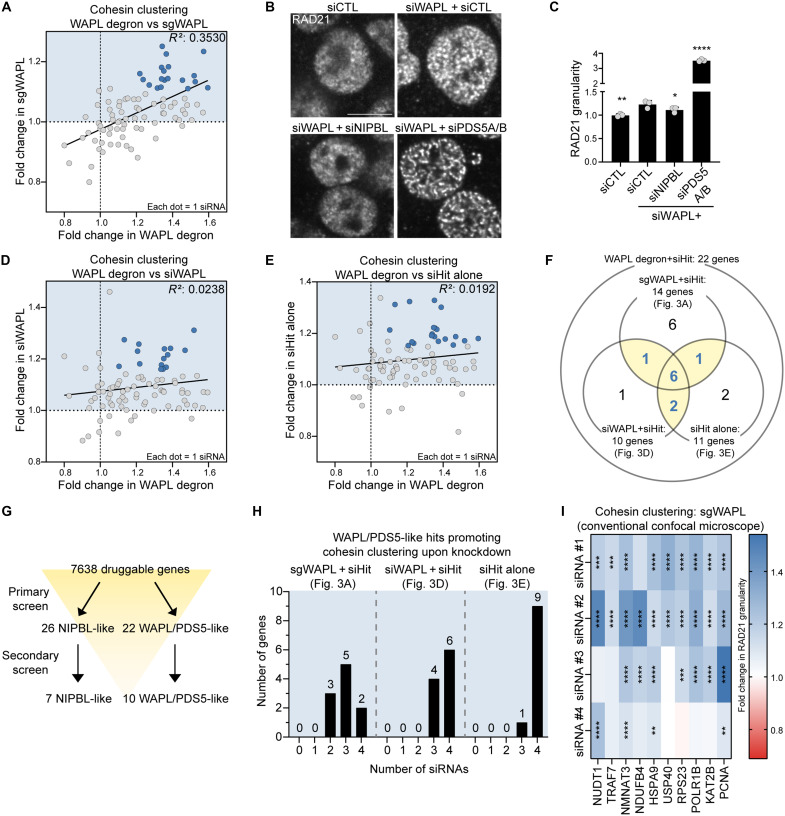
Secondary screens of WAPL/PDS5-like hits. (**A**, **D**, and **E**) Scatter plot showing relationships between fold changes in RAD21 granularity from the primary screen using WAPL degron and the secondary screens using WAPL KO (A), siRNA targeting WAPL (D), and siRNA targeting the hit alone (E). Data points represent individual siRNAs targeting one of 22 WAPL/PDS5-like hits (four siRNAs per hit). siRNAs passing a threshold [fold change >1.1 in WAPL KO (A), >1.15 in codepletion with siWAPL (D) or in siRNA targeting hit alone (E), and >1.1 in WAPL degron] are shown as blue circles. Simple linear regression line is included [*P* < 0.0001 (A), *P* = 0.1511 (D), and *P* = 0.1984 (E)]; *R*^2^ indicated. (**B**) RAD21 immunostaining of HCT116 cells transfected with nontargeting (CTL) siRNA, WAPL siRNA, or cotransfected with WAPL and NIPBL or PDS5A/B siRNAs. Scale bar, 10 μm. (**C**) Quantification of RAD21 granularity from (B). *P < 0.05, **P < 0.01, and ****P < 0.0001 (ANOVA with Dunnett’s test compared to siCTL + siWAPL). *n* = 3. Error bars denote mean + SD. (**F**) Venn diagrams summarizing the overlap of hits identified in the primary screen and the three secondary screens. Numbers of overlapping hits are labeled. (**G**) Screening workflow summarizing primary and secondary screens for identifying 7 NIPBL- and 10 WAPL/PDS5-like hits. (**H**) Numbers of genes with zero to four siRNAs showing increased RAD21 granularity in secondary screens for 10 WAPL/PDS5-like hits [siRNAs in light-blue area of (A), (D), and (E)]. (**I**) Heatmap of RAD21 granularity fold change for four siRNAs (columns) targeting 10 WAPL/PDS5-like hits (rows) in WAPL KO cells. Data were obtained using a conventional confocal microscope. Color intensity indicates the magnitude of fold change. **P < 0.01, ***P < 0.001, and ****P < 0.0001 (ANOVA with Dunnett’s test compared to siCTL).

We first measured RAD21 granularity upon knockdown of 26 NIPBL-like hits using individual siRNAs (4/gene) in WAPL KO cells (fig. S3A; *n* = 3). Eight siRNAs targeting seven genes resulted in the marked reduced cohesin clustering in both WAPL KO and degron cells using a stringent threshold (Fig. 2, F and G, highlighted in red circles; and table S2). Further analysis showed additional siRNAs targeting each of the seven genes reduced clustering, albeit with smaller effect sizes (Fig. 2H and table S2). Moreover, we confirmed these cohesin clustering phenotypes using conventional, nonautomated confocal microscopy, and at least two siRNAs targeting each gene attenuated cohesin clustering (Fig. 2I). This approach also ruled out potential bias introduced by automated high-content imaging. These genes included PHC2 (polyhomeotic homolog 2), a component of cPRC1 (canonical polycomb repressive complex 1) (*33*), and BCAS2 (breast carcinoma amplified sequence 2), an mRNA splicing factor (*34*, *35*). Next, we assessed the 22 WAPL/PDS5-like hits using individual siRNAs (4/gene; fig. S3B; *n* = 3); knockdown of 14 genes increased cohesin clustering in both WAPL KO and degron cells (Fig. 3A, highlighted in blue circles, and table S2).

As an additional test of WAPL/PDS5-like hits, we used siRNA-mediated WAPL knockdown. As expected, WAPL knockdown via siRNA resulted in weaker WAPL depletion and cohesin clustering than in the degron cells (Figs. 1, B and C, and 3B); however, siRNA-induced cohesin clustering was still suppressed by NIPBL knockdown and significantly enhanced by PDS5A/B knockdown (Fig. 3, B and C). Using the WAPL siRNA-treated cells, we tested the 22 WAPL/PDS5-like hits with individual siRNAs (4/gene; fig. S3C; *n* = 3) and found 10 genes whose knockdown increased clustering in both WAPL degron and WAPL siRNA cells (Fig. 3D, highlighted in blue circles, and table S2).

Next, we determined whether depletion of WAPL/PDS5-like hits induced cohesin clustering alone (without codepletion of WAPL). We tested the 22 WAPL/PDS5-like hits using individual siRNAs (4/gene; fig. S3D; *n* = 3), and found 11 genes whose knockdown increased clustering (Fig. 3E, highlighted in blue circles, and table S2).

We overlapped the results from the three series of experiments (WAPL KO, WAPL siRNA, the hit siRNA alone) and identified 10 genes whose knockdown consistently increased cohesin clustering in at least two conditions (Fig. 3, F and G, and table S2). Each gene was supported by multiple siRNAs that increased clustering across each of these three assays, albeit with smaller effect sizes (Fig. 3H and table S2). Cohesin clustering phenotypes for these 10 genes in WAPL KO cells were further confirmed using conventional confocal microscopy (Fig. 3I). At least two siRNAs targeting each gene promoted cohesin clustering in this assay.

We noticed that several NIPBL-like and WAPL/PDS5-like hits identified across the three experiments are components of multi-protein complexes. For example, BCAS2, a NIPBL-like hit, is a part of an mRNA splicing complex with PRPF19 (pre-mRNA processing factor 19) and CDC5L (cell division cycle 5 like) (*34*, *35*), but PRPF19 and CDC5L knockdown did not significantly affect clustering in the primary screening efforts (fig. S4A). PHC2, another NIPBL-like hit, is a cPRC1 component; depletion of other cPRC1 members, PCGF2 (polycomb group RING finger protein 2) and PCGF4, also attenuated clustering (fig. S4B). Last, NDUFB4 (NADH:ubiquinone oxidoreductase subunit B4), a WAPL/PDS5-like hit, is a subunit of mitochondrial complex I, and knockdown of several complex I components promoted clustering in the primary screen (fig. S4C). Together, we identified and prioritized seven NIPBL-like and 10 WAPL/PDS5-like proteins that modulate cohesin clustering from our secondary screen effort, some of which may act as part of protein complexes (table S2).

### Pharmacological and genetic validation of screening hits

To orthogonally validate the screening hits modulating cohesin clustering, we first tested if targeting hits with well-established small-molecule inhibitors can also modulate cohesin clustering. To inhibit NUDT1 (nudix hydrolase 1), PCNA (proliferating cell nuclear antigen), and NDUFB4 function, we used TH287, T2AA (T2 amino alcohol), and rotenone, respectively (*36*–*38*). In each case, target inhibition was confirmed, and treatment increased cohesin clustering in WAPL degron cells, consistent with the effects of siRNA-mediated depletion (fig. S5, A to F). KAT2B (lysine acetyltransferase 2B) was also targeted with three independent inhibitors (*39*–*41*), all of which produced a similar increase in cohesin clustering in WAPL degron cells (fig. S5G).

We then sought to additionally validate our results by performing CRISPR-based KO studies. Filtering the 17 candidates for nonessential genes left 12 candidates. We tested three NIPBL-like and three WAPL/PDS5-like hits of these 12 candidates. Among these, two NIPBL-like hits [CAND1 (cullin associated and neddylation dissociated 1) and BCAS2] and two WAPL/PDS5-like hits [NUDT1 and HSPA9 (heat shock protein family A (Hsp70) member 9)] were efficiently depleted by sgRNAs, whereas depletion of PHC2 (NIPBL-like) and USP40 (WAPL/PDS5-like) could not be achieved (fig. S5H). Loss of CAND1 or BCAS2 reduced cohesin clustering, while loss of NUDT1 or HSPA9 increased clustering, consistent with the siRNA results (fig. S5I). These pharmacological and CRISPR-based KO experiments validate a subset of screening hits as modulators of cohesin clustering.

### Cohesin clustering hits regulate TAD-TAD interaction

**Fig. 4.**
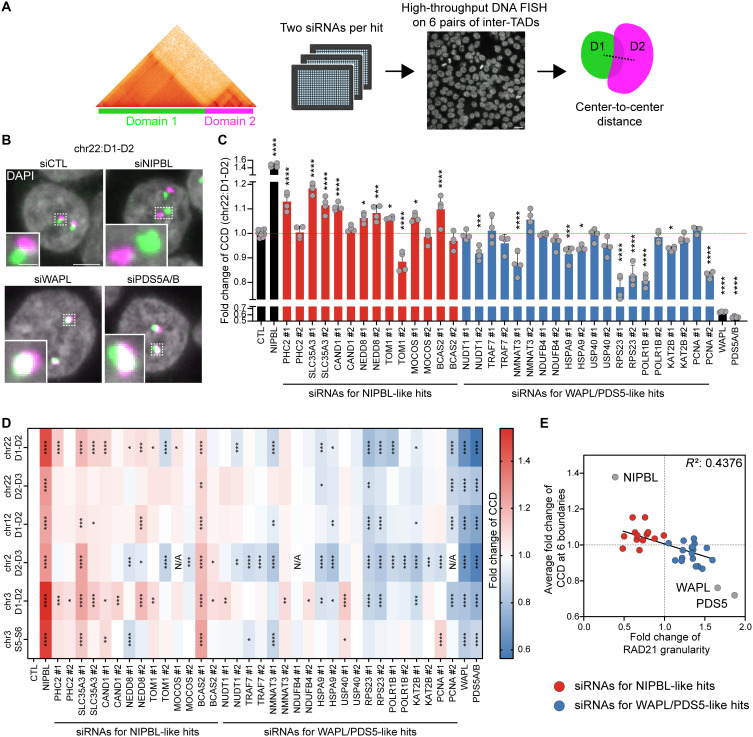
NIPBL- and WAPL/PDS5-like hits affect genome folding. (**A**) Workflow for DNA FISH experiments and analysis at two consecutive TADs [domain 1 (D1) and domain 2 (D2)]. Two siRNAs per hit were tested across six pairs of consecutive TADs using HiDRO. TAD-TAD interactions were quantified by measuring the center-to-center distance (CCD) between FISH signals. Scale bar, 10 μm. (**B** and **C**) DNA FISH for D1 and D2 on chromosome 22 (33.0 to 36.1 Mb) in HCT116 cells. (B) Representative FISH images of cells transfected with nontargeting siRNA or siRNAs targeting NIPBL, WAPL, and PDS5A/B. DNA was stained with DAPI (gray). Scale bars, 5 μm (nucleus) and 1 μm (magnified image). (C) Quantification of TAD-TAD interaction by measuring CCD upon knockdown of NIPBL- and WAPL/PDS5-like hits. Two siRNAs were tested per hit. The mean of nontargeting (CTL) siRNA control is indicated by red dotted line. *P < 0.05, ***P < 0.001, and ****P < 0.0001, as determined by ANOVA followed by Dunnett’s test to compare conditions to nontargeting siRNA. *n* > 4. Error bars denote mean + SD. (**D**) Heatmap showing average fold change in CCD across six TAD pairs (columns) for siRNAs targeting NIPBL- and WAPL/PDS5-like factors (rows). Color intensity indicates the magnitude of fold changes in CCD. *n* > 4. *P < 0.05, **P < 0.01, ***P < 0.001, and ****P < 0.0001, as determined by ANOVA followed by Dunnett’s test to compare conditions to nontargeting siRNA. Nontested conditions are indicated as not available (N/A). (**E**) Scatter plot showing relationship between RAD21 granularity fold change under WAPL degron condition (*x* axis) and CCD fold change from DNA FISH experiments (*y* axis). Fold change in CCD across six TAD pairs was averaged. Red and blue dots represent siRNAs targeting NIPBL- and WAPL/PDS5-like hits, respectively. Simple linear regression line is included (*P* < 0.0001), and *R*^2^ value is indicated.

Cohesin clustering measured by immunostaining is efficient, but does not directly measure genome folding. To address this, we used high-throughput DNA or RNA labeling with optimized Oligopaints (HiDRO), which we developed to efficiently conduct a large number of DNA fluorescence in situ hybridization (FISH) reactions. We previously used HiDRO to identify genes affecting the interaction between two adjacent TADs on chromosome 22 (33.0 to 36.1 Mb) (*28*), and we performed HiDRO on this same TAD pair following knockdown of screening hits. Inter-TAD interaction was assessed by measuring center-to-center distance (CCD) between the two TADs labeled with FISH probes (Fig. 4A). We first knocked down NIPBL, WAPL, or PDS5A/B and conducted DNA FISH using HiDRO. Consistent with our previous findings, NIPBL depletion increased, and WAPL or PDS5A/B depletion decreased the CCD (Fig. 4, B and C) (*28*). We then performed HiDRO upon knockdown of the seven NIPBL-like and 10 WAPL/PDS5-like hits confirmed in multiple assays, using two siRNAs for each gene that significantly suppressed in WAPL degron cells (NIPBL-like) or increased cohesin clustering in WAPL degron or wild type cells (WAPL/PDS5-like; *n* = 4; fig. S6). Efficient target depletion was confirmed for all siRNAs (fig. S7, A and B). We found that knockdown of multiple NIPBL- and WAPL/PDS5-like hits, respectively, increased or decreased CCD with at least one siRNA (Fig. 4C).

We extended this analysis to five additional pairs of adjacent TADs across chromosomes 2, 3, 12, and 22 (*42*); six NIPBL- and eight WAPL/PDS5-like hits consistently altered inter-TAD interactions across two or more TAD pairs with at least one siRNA (Fig. 4D and table S3). CCD changes and cohesin clustering changes correlated (Fig. 4E), suggesting several NIPBL-like hits behave similarly to NIPBL, and WAPL/PDS5-like hits similarly to WAPL and PDS5. Collectively, these experiments demonstrate successful identification of genome folding regulators via screening for cohesin clustering modulation.

### CTCF clustering distinguishes WAPL- and PDS5-like hits

**Fig. 5.**
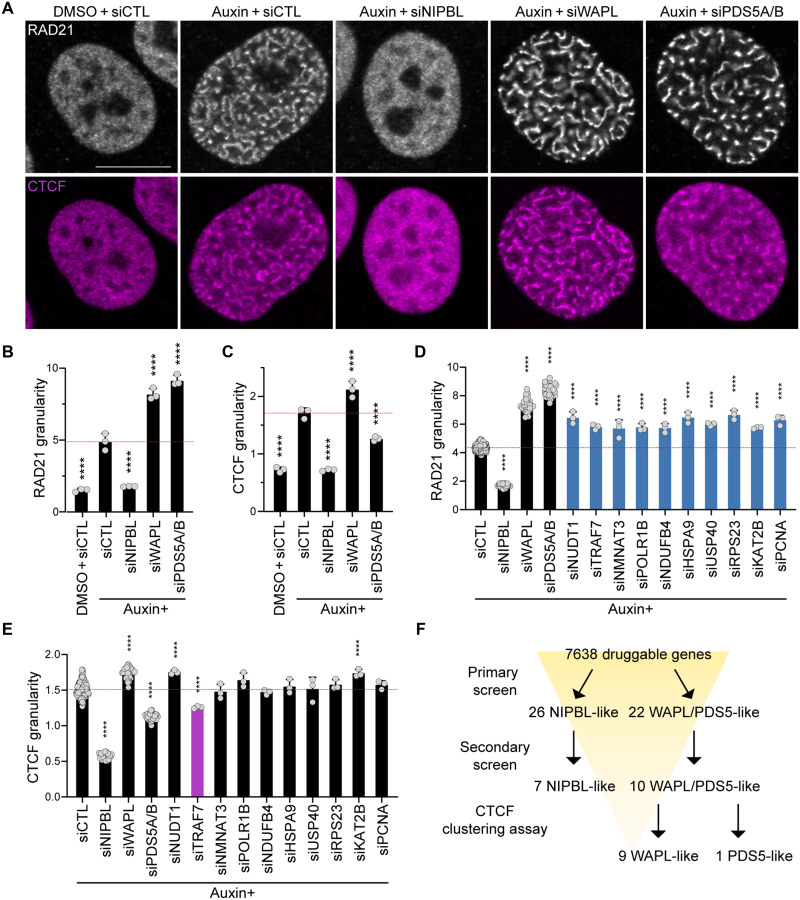
CTCF clustering distinguishes WAPL- from PDS5-like hits. (**A** to **C**) RAD21 and CTCF immunostaining of HCT116:WAPL-AID cells treated with DMSO or auxin and transfected with nontargeting (CTL) siRNA or siRNAs targeting NIPBL, WAPL, or PDS5A/B. Cells were treated with 1 μM auxin for 72 hours. (A) Representative images of RAD21 (gray) and CTCF (magenta) immunostaining. Scale bar, 10 μm. (B and C) Quantification of RAD21 (B) and CTCF (C) granularity. ****P < 0.0001, as determined by ANOVA followed by Dunnett’s test to compare conditions to nontargeting siRNA with auxin. *n* = 3. Error bars denote mean + SD. (**D** and **E**) Quantification of RAD21 (D) and CTCF (E) granularity in HCT116:WAPL-AID cells treated with auxin and transfected with siRNAs targeting WAPL/PDS5-like hits. Cells were treated with 1 μM auxin for 72 hours. ****P < 0.0001, as determined by ANOVA followed by Dunnett’s test to compare conditions to nontargeting siRNA with auxin. *n* > 3. Error bars denote mean + SD. (B to E) The mean of auxin treatment and nontargeting siRNA control is indicated by red dotted lines. (**F**) Screening workflow summarizing primary and secondary screens, along with the CTCF clustering assay, for identifying WAPL- and PDS5-like hits.

WAPL KO increases the number of cohesin-mediated chromatin loops (*9*, *17*) and CTCF stabilizes chromatin loops partly by directly interacting with cohesin (*43*). We found CTCF clusters in WAPL degron and NIPBL depletion suppressed this clustering, suggesting CTCF clustering is, at least in part, mediated by cohesin activity (Fig. 5, A and C). Additional WAPL depletion by siRNA enhanced both cohesin and CTCF clustering. In contrast, PDS5 depletion increased cohesin clustering but decreased CTCF clustering (Fig. 5, A to C). This suggests that PDS5 depletion may attenuate the probability of anchoring of chromatin loops at CTCF sites, consistent with PDS5 depletion decreasing the number of loops detected by Hi-C (see Discussion) (*9*, *44*). Given the distinct effects of WAPL and PDS5 depletion on CTCF clustering, we stratified WAPL/PDS5-like hits based their effect on CTCF clustering. For each of the 10 WAPL/PDS5-like hits, we selected the siRNA resulting in the strongest cohesin clustering in WAPL degron cells. We again confirmed efficient target depletion (fig. S7C) and assessed cohesin and CTCF clustering. Consistent with the primary screen, all siRNAs increased cohesin clustering (Fig. 5D). Notably, TRAF7 (TNF receptor associated factor 7) depletion decreased CTCF clustering while the other hits did not (Fig. 5E), suggesting that TRAF7, an E3 ubiquitin ligase, functions as a PDS5-like hit (Fig. 5F).

### Investigating mechanisms by which hits may regulate cohesin

**Fig. 6.**
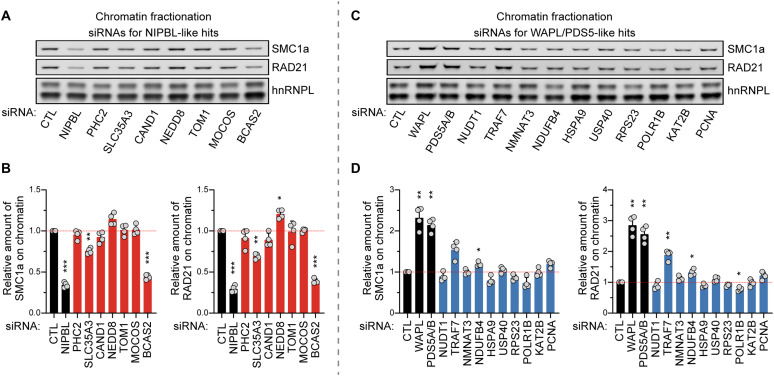
Chromatin-bound cohesin levels upon knockdown of screening hits. (**A** and **C**) Immunoblots of SMC1a and RAD21 from HCT116 cells transfected with siRNAs targeting 7 NIPBL-like hits (A) or 10 WAPL/PDS5-like hits (C). One biological replicate is shown; three additional replicates shown in fig. S8. hnRNPL was used as a loading control. (**B** and **D**) Quantification of SMC1a (left) and RAD21 (right) from (A) and (C) for NIPBL-like hits (B) or WAPL/PDS5-like hits (D). Band intensities normalized to hnRNPL. *P < 0.05, **P < 0.01, and ***P < 0.001, as determined by ANOVA followed by Dunnett’s test to compare to nontargeting (CTL) siRNA. *n* = 4. Error bars denote mean + SD.

Having established a series of NIPBL- and WAPL/PDS5-like factors that modulate cohesin clustering, we sought to understand straightforward mechanisms by which they may control cohesin. One potential mechanism to regulate cohesin clustering is by affecting cohesin levels on chromatin. As expected for a cohesin loader, NIPBL depletion reduced cohesin levels bound to chromatin (Fig. 6, A and B, and fig. S8A). Among the NIPBL-like hits, SLC35A3 (solute carrier family 35 member A3) and BCAS2 also decreased levels of cohesin on chromatin (Fig. 6, A and B, and fig. S8A). In contrast, knockdown of WAPL or PDS5A/B increased cohesin on chromatin, consistent with previous reports that their depletion increases cohesin residence time on chromatin (Fig. 6, C and D, and fig. S8B) (*9*, *26*). Among the WAPL/PDS5-like hits, TRAF7 and NDUFB4 increased chromatin-bound cohesin (Fig. 6, C and D, and fig. S8B). These data suggest that BCAS2, SLC35A3, TRAF7, and NDUFB4 may regulate cohesin clustering, at least in part, by modulating cohesin levels on chromatin.

A second potential mechanism to modulate cohesin clustering is by affecting cohesin protein levels. NIPBL knockdown decreased the amount of RAD21, suggesting that cohesin protein is stabilized when bound to chromatin (fig. S9, A and B). BCAS2 knockdown also reduced RAD21 protein levels, while the other hits did not decrease RAD21 levels (fig. S9, A and B). We further tested whether hits affected the abundance of key cohesin-associated factors: NIPBL, WAPL, and PDS5A. Among NIPBL-like hits, SLC35A3 and BCAS2 depletion decreased NIPBL levels (fig. S9, A and C). PHC2, CAND1, NEDD8 (NEDD8 ubiquitin like modifier), and TOM1 (target of myb1 membrane trafficking protein) depletion increased PDS5A levels (fig. S9, A and D). Among WAPL/PDS5-like hits, TRAF7 and PCNA knockdown reduced PDS5A levels (fig. S9, A and D). These data raise the possibility that some hits may regulate cohesin clustering by altering NIPBL or PDS5A protein levels. We also found several hits also affected WAPL protein abundance (fig. S9, A and E). However, since these hits modulated cohesin clustering in WAPL KO cells, they may regulate cohesin clustering through multiple mechanisms.

### Testing candidates for physical interaction with cohesin

**Fig. 7.**
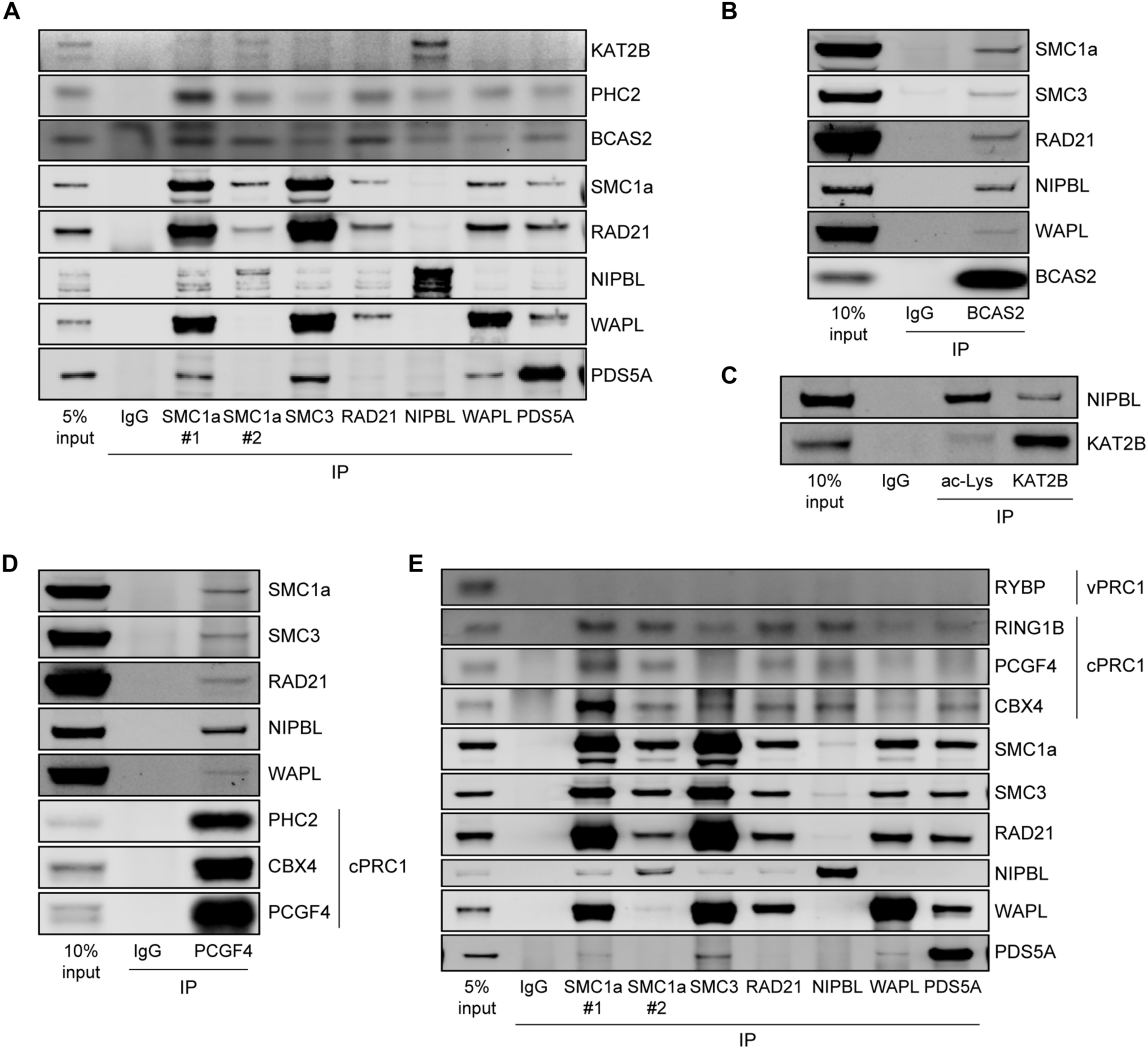
Select hits physically interact with cohesin and cohesin-associated factors. (**A**) IP of cohesin and cohesin-associated factors from HCT116 cells, followed by immunoblots analysis using the indicated antibodies to detect interactions with KAT2B, PHC2, and BCAS2. Two antibodies for SMC1a were used for IP. (**B**) Reciprocal IP of BCAS2, followed by immunoblot analysis using the indicated antibodies. (**C**) Reciprocal immunoprecipitation of acetylated protein and KAT2B, followed by immunoblot analysis using the indicated antibody. (**D** and **E**) Interaction between cPRC1 and cohesin/cohesin-associated factors. (D) IP of cPRC1 using PCGF4 antibody, followed by immunoblot analysis using the indicated antibodies. (E) IP of cohesin and cohesin-associated factors, followed by immunoblot analysis using antibodies against the cPRC1 components (RING1B, PCGF4, and CBX4) and the vPRC1 component (RYBP). Two antibodies for SMC1a were used for IP.

Last, we observed that many of our screening hits were predicted to localize to the nucleus, leading us to test whether they interact with cohesin and its associated factors. After establishing conditions to solubilize the cohesin complex (fig. S10, A and B), we performed coimmunoprecipitation (co-IP) assays in HCT116 cells. PHC2 and BCAS2 co-IP’ed with all cohesin complex components and associated factors tested, while KAT2B interacted specifically with NIPBL (Fig. 7A). In contrast, PCNA, POLR1B (RNA polymerase I subunit B) and CAND1, all nuclear-localized, did not co-IP with cohesin complex components (fig. S10, C and D). The interaction of PHC2, BCAS2, and KAT2B with NIPBL was also detected in HEK293T cells (fig. S10E). Reciprocal co-IP experiments confirmed that BCAS2 and KAT2B interact with NIPBL, with BCAS2 also interacting, albeit more weakly, with cohesin components and WAPL (Fig. 7, B and C). KAT2B is an acetyltransferase, and we found that NIPBL was co-IPed with a pan-acetyl-lysine antibody. However, KAT2B depletion did not affect the ability of the pan-acetyl-lysine antibody to IP NIPBL (fig. S11, A and B) or reduce the level of SMC3 (structural maintenance of chromosomes 3) acetylation, a key modification that regulates cohesin function (fig. S11C) (*45*, *46*). These data suggest KAT2B may regulate cohesin through acetylation of multiple proteins, including those other than NIPBL or SMC3. Last, we tested if cohesin interacts with cPRC1. cPRC1 was co-IPed using an antibody against PCGF4 together with NIPBL, and, to a lesser extent, with cohesin and WAPL (Fig. 7D). Reciprocal co-IP confirmed cPRC1 components, PCGF4, CBX4 (Chromobox 4), and RING1B (RING Finger Protein 1B), co-IPed with cohesin and associated factors (Fig. 7E). This interaction is specific to cPRC1; RYBP (RING1 and YY1 binding protein), a component of vPRC1 (variant PRC1), did not interact with cohesin and associated factors (Fig. 7E). Collectively, the co-IP experiments indicate that some nuclear proteins identified in the cohesin clustering screen and related efforts may regulate cohesin clustering and genome folding through direct interactions with cohesin or its associated factors.

## DISCUSSION

The cohesin complex is a key molecular machine regulating genome folding; however, identifying upstream regulators of cohesin-mediated genome folding has remained challenging, partly due to the limited high-throughput capacity of genomic techniques such as Hi-C. Here, we established an imaging-based genetic screening platform measuring cohesin clustering. We screened 7638 druggable genes and identified modulators of cohesin clustering under partial WAPL degradation. Depletion of known cohesin regulators, including NIPBL, WAPL, and PDS5A/B, suppressed or enhanced this phenotype, indicating that regulators of cohesin loading, loop extrusion activity, and unloading can be identified using this screening approach. Through screening and orthogonal experiments, we identified seven NIPBL-, nine WAPL- and one PDS5-like factors.

Our study provides a framework for understanding how upstream pathways regulate specific steps of cohesin function. Long-standing questions in the field have focused on the identity of molecular determinants of cohesin loading, loop extrusion, unloading, and CTCF-anchored loop stabilization. More broadly, it has remained unclear if and how these steps are controlled by diverse pathways. By defining NIPBL-like, WAPL-like, and PDS5-like categories using quantitative phenotypes and orthogonal validation, we connect pathways such as mitochondrial metabolism, epigenetic silencing, and post-translational modification to distinct steps of the cohesin life cycle. This framework immediately generates testable hypotheses about how these pathways control genome folding. Thus, the value of this screen extends beyond initial hit discovery, providing a resource that enables subsequent work to uncover the myriad mechanisms that mediate cohesin activity, genome folding and the implications for cellular function.

We previously developed and used HiDRO to screen 3083 druggable genes to identify regulators of interactions between two adjacent TADs (*28*). The approach identified GSK3A as a regulator of cohesin and genome folding. Cohesin clustering provides an orthogonal strategy to identify cohesin regulators, but each approach has important strengths to consider. Cohesin clustering reflects global cohesin activity, whereas HiDRO examines inter-TAD interactions at specific genomic loci. Cohesin is dynamically loading, extruding, and unloading; thus, TAD-TAD interaction frequency by DNA FISH can be variable, potentially reflecting genome organization dynamics (*47*, *48*). In contrast, cohesin clustering is a coarser phenotype and less likely to reflect this variability. In turn, fewer cells are required to determine the effect of any perturbation. A limitation of the clustering assay is the sensitized background with partial WAPL depletion. Moreover, cohesin clustering is not a direct measurement of cohesin activity. Despite these limitations, cohesin clustering and TAD-TAD interaction frequency offer orthogonal and complementary strengths to study cohesin activity.

Our study discovered several intriguing factors as potential cohesin regulators. First, BCAS2 was identified as a NIPBL-like factor; BCAS2 depletion suppressed cohesin clustering and reduced TAD-TAD interaction frequency. Previous studies identified BCAS2 as a component of a splicing factor complex with PRPF19/CDC5L (*34*, *35*); however, PRPF19 and CDC5L were not identified in our screen, suggesting a possible splicing-independent role for BCAS2 in cohesin regulation. Notably, BCAS2 depletion results in reduced levels of chromatin-bound cohesin. Future studies can investigate if BCAS2 regulates cohesin loading and loop extrusion via the interaction with NIPBL we observed.

A second interesting hit is NDUFB4, a subunit of mitochondrial complex I, which was identified as a WAPL-like factor. Multiple components of complex I were identified as enhancers in the primary screen, and pharmacological inhibition of complex I with rotenone increased cohesin clustering. Moreover, NDUFB4 depletion increased chromatin-bound cohesin, indicating this complex limits cohesin loading. Complex I is known to function in producing ATP, NAD+ (*49*, *50*), and reactive oxygen species (*51*). A previous study reported ATP depletion by oligomycin treatment, which inhibits mitochondrial complex V, impairs chromatin loop formation (*52*). In contrast, our findings suggest mitochondrial complex I functions in a WAPL-like manner, suggesting distinct and possibly opposing roles of different mitochondrial complexes on cohesin activity.

Third, KAT2B acetyltransferase was identified as a WAPL-like factor; KAT2B is a lysine acetyltransferase known to acetylate both histone and nonhistone substrates (*53*). Pharmacological inhibition of KAT2B with three small molecules phenocopied the effects of KAT2B genetic depletion on cohesin clustering, indicating that acetyltransferase activity is required for KAT2B-mediated regulation of cohesin. NIPBL and SMC3 seem unlikely to be the relevant substrates, and identifying the acetylation target and elucidating the molecular mechanisms underlying KAT2B-cohesin regulation will be an exciting future direction.

Last, a fourth notable hit is PHC2, a component of cPRC1. Similar to NIPBL loss, PHC2 depletion suppressed cohesin clustering and reduced TAD-TAD interaction frequency. PRC1 complexes, which catalyze histone mono-ubiquitination, are classified into cPRC1 and vPRC1 subtypes (*33*). PHC2 is specific to cPRC1 and multiple cPRC1 components interact with cohesin, whereas the vPRC1-specific component RYBP did not. PRC1 silences developmental genes and promotes long-range chromatin compaction into Polycomb bodies. Moreover, cohesin controls PRC1 chromatin binding and PRC1-dependent chromatin compaction (*54*, *55*), but it remains unclear whether PRC1 reciprocally regulates cohesin activity. A next step would be investigating whether (i) cPRC1 ubiquitination activity is required for cohesin regulation, (ii) cPRC1 influences cohesin through chromatin compaction, or (iii) cPRC1 directly affects cohesin via protein-protein interactions.

Only a subset of screening hits affected cohesin levels on chromatin, indicating that cohesin clustering can be controlled via mechanisms beyond loading. For example, it is possible that some hits specifically modulate the loop extrusion activity of cohesin, rather than its levels on chromatin. Whereas WAPL depletion increases cohesin residence time, PDS5 depletion increases both residence time and extrusion rates (*21*). Upon mitotic release, PDS5 depletion induces cohesin clustering more rapidly than WAPL depletion (*21*), suggesting that cohesin clustering may reflect enhanced extrusion activity. Alternatively, factors that bind chromatin may alter cohesin translocation indirectly, possibly by acting as physical obstacles to extrusion. For example, the MCM complex physically constrains cohesin (*56*). MCM3 (minichromosome maintenance complex component 3) directly interacts with cohesin via its YDF motif (*43*, *56*), and loss of MCM3 enhanced cohesin clustering in the primary screen (table S1). Thus, it appears that different steps of the cohesin life cycle may be controlled by different factors to provide precise control of loop extrusion.

Our study also demonstrated CTCF and cohesin clustering can be unlinked. PDS5 is a WAPL cofactor facilitating cohesin unloading, and our data also suggests PDS5 promotes CTCF-mediated cohesin stalling. PDS5A/B and WAPL codepletion decreases CTCF clustering to WAPL depletion alone. These data are consistent with studies showing PDS5A/B depletion increases loop size, but decreases the number of CTCF-anchored loops (*9*). Several molecular mechanisms can explain how PDS5 regulates cohesin and CTCF-mediated loop stabilization. First, PDS5 competes with NIPBL for cohesin binding (*15*, *57*). While NIPBL stimulates cohesin loop extrusion activity, this activity likely needs to be attenuated at loop anchors. Given that PDS5A and PDS5B binding is enriched at CTCF sites (*44*, *58*), PDS5 may compete with NIPBL for cohesin binding to halt loop extrusion at CTCF-bound sites and thereby stabilize loops. Second, cohesin acetylation is required to maintain TAD boundary fidelity (*25*). ESCO1 (establishment of sister chromatid cohesion N-acetyltransferase 1) acetylates SMC3 and loss of ESCO1 reduces TAD insulation, resembling CTCF loss (*25*, *59*, *60*). Moreover, PDS5 is required for ESCO1-dependent SMC3 acetylation (*34*, *61*, *62*). Therefore, it is possible PDS5 may facilitate cohesin acetylation in an ESCO1-dependent manner to promote stalling at CTCF sites. Third, PDS5A physically interacts with CTCF (*63*). The PDS5-binding region on CTCF contains amino acid sequences similar to the known PDS5-binding domains of WAPL (*63*). This opens the possibility PDS5 competes with WAPL for CTCF binding to protect cohesin from WAPL-mediated unloading at CTCF sites. These nonmutually exclusive mechanisms may explain how PDS5 stabilizes CTCF-anchored loops. Two recent preprint manuscripts support the first model, showing that PDS5 stabilizes CTCF-anchored loops and TAD boundaries by limiting cohesin-NIPBL complexes (*21*, *22*). It will be informative to test whether CTCF clustering observed in PDS5-depleted cells is dependent on NIPBL. Our experiments identified TRAF7 as a PDS5-like factor, and depletion of TRAF7 phenocopied the effects of PDS5A/B depletion in WAPL degron cells, resulting in reduced CTCF clustering. Future studies should explore whether TRAF7, an E3 ubiquitin ligase, modulates PDS5 activity and determine which specific PDS5-mediated processes described above are affected by TRAF7.

Our study identified several potential regulators of cohesin involved in diverse cellular and molecular functions. In addition, our experiments and validation studies suggest that different aspects of the cohesin life cycle are likely to be regulated by different factors. Broadly, a central question across multiple fields has focused on how cohesin-mediated genome structure is controlled. Identification of and in-depth dissection of the molecular factors that govern cohesin activity will undoubtedly elucidate how genome structure contributes to cellular function. Collectively, our resource opens multiple avenues for future mechanistic work into cohesin regulation and broader implications for genome folding in health and disease.

## MATERIALS AND METHODS

### Cell culture

HCT116 and Lenti-X HEK293T cells were purchased from American Type Culture Collection (ATCC, no. CRL-247) and Takara (no. 632180), respectively. HCT116:WAPL-AID cells were generated as described previously (*28*). Cells were maintained at 37°C in 20% O_2_ and 5% CO_2_. HCT116 cells were cultured in McCoy’s 5A medium (Invitrogen, no. 6600082) supplemented with 10% fetal bovine serum (FBS), 1× Glutamax (Invitrogen, no. 35050061) and 1% penicillin-streptomycin (Invitrogen, no. 15140122). Lenti-X HEK293T cells were cultured in Dulbecco’s modified Eagle’s medium (Invitrogen, no. 11995065) supplemented with 10% FBS and 1% penicillin-streptomycin. 5-Ph-IAA (modified auxin; a gift from M. Kanemaki’s laboratory) was treated at indicated concentrations and times, as shown in the figure and figure legends.

### Primary screen using pooled siRNA

Poly-d-lysine–coated 384-well plates (Perkin Elmer, no. 6057300) in which 1.25 pmole of siRNA (Dharmacon) was preseeded were ordered from the Harvard Medical School ICCB-Longwood Screening Facility. Each plate contained ~280 assay wells, with the outer wells used for controls or left empty. Control siRNAs (Dharmacon) were manually seeded: nontargeting control (no. D-001210-05-20), NIPBL (no. J-012980-08-0010), WAPL (no. L-026287-01-0010), PDS5A (no. M-014071-02-0005), and PDS5B (no. M-010362-00-0005). RNAiMAX transfection reagent (Invitrogen, no. 13778075) was diluted in Opti-MEM reduced serum medium (Invitrogen, no. 31985070) at 0.1 μl of RNAiMAX per 8.9 μl of Opti-MEM per well. The mixture was applied to siRNA using the Matrix WellMate (Invitrogen), followed by incubation for 20 min at room temperature. HCT116:WAPL-AID cells were trypsinized and diluted in auxin-containing medium. A total of 6000 cells were seeded in each well using the Matrix WellMate to achieve a final auxin concentration of 1 μM. To get even cell distribution, the plates were centrifuged at 500 rpm for 1 min at room temperature. After 24 hours, the medium was removed using an aspiration wand (V&P Scientific, no. VP186L-1). Fresh medium containing 1 μM auxin was added using the Matrix WellMate. After 24 hours, additional medium with auxin was added to achieve a final concentration of 2 μM. After another 24 hours, cells are fixed and immunostained.

For immunostaining, the medium was removed from 384-well plates and freshly prepared 4% paraformaldehyde (Electron Microscopy Sciences, no. 15710) in phosphate-buffered saline (PBS) was added in each well. Cells were incubated with fixative for 12 min at room temperature. Fixed cells were washed twice with PBS for 10 min, followed by permeabilization with 0.5% Triton X-100 in PBS for 10 min. Cells were washed twice with PBS for 10 min, and blocked with 3% bovine serum albumin (Sigma-Aldrich, no. A9647) in PBS with 0.1% Tween-20 (PBST) for 1 hour at room temperature. RAD21 antibody (1:500, Sigma-Aldrich, no. 05-908) diluted in blocking solution was added, and cells were incubated for 24 hours at 4°C. Cells were washed three times with PBST for 10 min and stained with anti-mouse Alexa Fluor 555 secondary antibody (1:1000, Invitrogen, no. A32727) and 4′,6-diamidino-2-phenylindole (DAPI; 1 μg/ml, Sigma-Aldrich, no. D5942) in blocking solution for 4 hours at room temperature. Cells were washed three times with PBST for 10 min and twice with PBS for 10 min. Plates were stored in PBS at 4°C until imaging.

For imaging, PBS was removed and imaging buffer [50 mM tris-HCl (pH 8.0), 10 mM NaCl, 10% glucose, catalase (0.1 mg/ml; Sigma-Aldrich, no. C3155), and glucose oxidase (0.37 mg/ml; Sigma-Aldrich, no. G2133)] was added. DAPI and RAD21 images were acquired using a Molecular Devices ImageXpress Micro 4 high-content confocal microscope with a 0.45-μm pinhole and a 1.4–numerical aperture (NA) 60× water-immersion objective. Best focus projections of *z*-stacks (13 images, 1.3 μm spacing) were automatically generated in MetaXpress.

For image analysis, CellProfiler software (version 4.2.1) was used. Individual nuclei were first segmented from DAPI images using two-class Otsu thresholding method. To quantify cohesin clustering, RAD21 staining granularity was measured using “MeasureGranularity” module. This module analyzes the size of clusters in an image by progressively removing objects using a structural element of a specific size and calculating the signal change to assess the distribution of object sizes. In the primary screen, RAD21 granularity was analyzed with structuring element radius of 60 pixels and a range of granular spectrum of 5. The granularity measurement for the second instance of the granularity spectrum was obtained. For each plate, the median RAD21 granularity per well was obtained and *z* scores were calculated for each well. Genes were chosen as NIPBL-like hits if they had *z* score below −2.1 in both replicates or below −2.45 in any replicate. Genes were chosen as WAPL/PDS5-like hits if they had *z* score above 2.45 in both replicates or above 2.9 in any replicate. This identified 80 NIPBL-like and 38 WAPL/PDS5-like hits.

### De-pooled siRNA validation

Poly-d-lysine–coated 384-well plates pre-seeded with 1.25 pmole of four individual siRNAs targeting primary screening hits were obtained from the Harvard Medical School ICCB-Longwood Screening Facility. Cell culture, siRNA transfection, immunostaining, imaging, and image analysis were conducted as described for primary screen. Each siRNA was tested in triplicates. Nontargeting siRNAs were manually added to 112 wells, and NIPBL and WAPL-targeting siRNAs were manually added to 12 wells each. For each plate, the median RAD21 granularity per well was obtained and the RAD21 granularity fold change relative to the nontargeting siRNA control was calculated for each siRNA.

Primary hits in this experiment were prioritized based on their effect size on cohesin clustering as follow: for NIPBL-like hits, the most effective siRNA decreased clustering by >20% and the second best by >10%; for WAPL/PDS5-like hits, the most effective siRNA increased clustering by >30% and the second best by >10%. Using this criteria, 26 NIPBL-like and 22 WAPL/PDS5-like hits were prioritized.

### sgRNA cloning and lentivirus production

A previous publication identified two residues in WAPL (M1116 and Y725) essential for its function (*20*). sgRNAs targeting these residues were individually cloned into the lentiCRISPRv2-puro vector (Addgene, no. 52961) at the BsmB I sites. sgRNAs targeting screening hits were cloned into the same vector. Primer sequences are listed in table S3. Lentiviruses were produced in Lenti-X HEK293T cells using standard packaging plasmids (psPAX2: Addgene, no. 12260 and pMD2.G: Addgene, no. 12259) along with plasmids expressing sgRNA targeting WAPL M1116 and Y725, following manufacturer instructions. Plasmid transfections were performed using Lipofectamine 3000 (Invitrogen, no. L3000015) according to manufacturer instructions. Lentivirus-containing media were collected after 24 hours, filtered through a 0.45 μm polyethersulfone membrane (Genesee Scientific, no. 25-246).

### Secondary screen

For secondary screen with WAPL KO cells, wild-type HCT116 cells were transduced with lentivirus to express sgRNAs targeting WAPL M1116 and Y725 in the presence of polybrene (5 μg/ml; Sigma-Aldrich, no. TR-1003-G) for 24 hours. Infected cells were selected with puromycin (1 μg/ml) for 2 days. On the day of transfection, 1.25 pmole of siRNAs targeting screening hits were seeded in poly-d-lysine–coated 384-well plates and incubated with RNAiMAX diluted in Opti-MEM reduced serum medium for 20 min at room temperature. The puromycin-selected 6000 cells were seeded to the wells. After 3 days, cells were fixed and immunostained.

For secondary screen with siRNA targeting WAPL, 1.25 pmole of siRNAs targeting screening hits were seeded in poly-d-lysine–coated 384-well plates. A master mix of WAPL-targeting siRNA and RNAiMAX transfection reagent was prepared in Opti-MEM reduced serum medium. Aliquots of master mix were added to the wells so that 1.25 pmole of WAPL-targeting siRNA was added in each well. The mixture was incubated for 20 min at room temperature. A total of 2500 wild-type HCT116 cells were seeded per well. After 3 days, cells were fixed and immunostained.

For secondary screen targeting screening hit alone, 1.25 pmole of siRNAs targeting screening hits were seeded in poly-d-lysine–coated 384-well plates. RNAiMAX transfection reagent diluted in Opti-MEM reduced serum medium was added to the wells. After incubation for 20 min at room temperature, 2500 wild-type HCT116 cells were seeded per well. After 3 days, cells were fixed and immunostained.

For all secondary screens, nontargeting siRNA was added to 44 wells, and NIPBL, WAPL, and PDS5A/B-targeting siRNAs were added to 12 wells each as controls. Immunostaining, imaging, and image analysis were performed as described for the primary screening. Each siRNA was tested in triplicates.

For each plate, the median RAD21 granularity per well was obtained and the RAD21 granularity fold change relative to the nontargeting siRNA control was calculated for each siRNA. In the secondary screen with WAPL KO cells, genes were chosen as NIPBL-like hits if any siRNA produced a fold change <0.87 in WAPL KO and < 0.9 in WAPL degron cells (Fig. 2F), and as WAPL/PDS5-like hits if any siRNA produced a fold change >1.1 in both WAPL KO and WAPL degron cells (Fig. 3A). In the secondary screen with siRNA targeting WAPL, genes were chosen as hits if any siRNA produced a fold change >1.15 in codepletion with siWAPL and >1.1 in WAPL degron cells (Fig. 3D). In secondary screen targeting the screening hit alone, genes were chosen as hits if any siRNA produced a fold change >1.15 in WAPL KO and >1.1 in WAPL degron cells (Fig. 3E). siRNA catalog information can be found in table S2.

### Immunostaining for imaging with conventional confocal microscopy

For experiments (Figs. 1C, 2I, 3I, and 5A, and fig. S5), the chamber slide (Cellvis, no. C18SB-1.5H) were coated with poly-d-lysine. siRNA was mixed with RNAiMAX transfection reagent in Opti-MEM reduced serum medium and incubated for 20 min at room temperature. The siRNA mixture was then applied to the chamber slide, and cells were seeded on top. Cells were treated with 1 μM auxin accordingly as needed. For immunostaining, cells were fixed with 4% paraformaldehyde in PBS for 12 min at room temperature, followed by two washes with PBS for 10 min each. Cells were permeabilized with 0.5% Triton X-100 in PBS for 10 min at room temperature and then washed twice with PBS for 10 min. Cells were blocked with 3% BSA PBST for 1 hour and incubated with antibody diluted in blocking solution for 24 hours at 4°C. After incubation, cells were washed three times with PBST for 10 min each and stained with secondary antibody and DAPI (1 μg/ml; Sigma-Aldrich, no. D5942) diluted in blocking solution for 4 hours at room temperature. After washing three times with PBST and twice with PBS for 10 min each, imaging buffer [50 mM tris-HCl (pH 8.0), 10 mM NaCl, 10% glucose, catalase (0.1 mg/ml; Sigma-Aldrich, no. C3155), and glucose oxidase (0.37 mg/ml; Sigma-Aldrich, no. G2133)] was added. Images were obtained with a Leica SP8 confocal microscope using ×63/1.40 oil objective. For image analysis, nuclei were segmented as described for the primary screen. RAD21 granularity was analyzed with structuring element radius of 60 pixels and a range of granular spectrum of 5. The granularity measurement for the second instance of the granularity spectrum was obtained.

### RAD21 and CTCF coimmunostaining and analysis for screening hits

siRNAs (1.25 pmole) targeting screening hits were seeded in poly-d-lysine–coated 384-well plates and were transfected into HCT116:WAPL-AID cells, as described for the primary screen. Cell culture, immunostaining, and imaging were conducted following the primary screen protocol, with one modification: Images were acquired with narrower *z* spacing (15 images, 0.3 μm spacing) to achieve better resolution along *z* plane. Projection images were generated using the “Best focus projections” module in MetaXpress. For quantification of cohesin and CTCF clustering, RAD21 and CTCF staining granularity were measured using the “MeasureGranularity” module with structuring element radius of 60 pixels and a range of granular spectrum of 5. The granularity measurement for the second instance of the granularity spectrum was used for analysis. The median RAD21 and CTCF granularity per well were obtained, and fold changes were calculated for each well. Each siRNA was tested in triplicates.

### HiDRO

Oligos were designed and synthesized as described previously (*28*). Coordinates of domains labeled by probes in DNA FISH experiments are available in table S3. HiDRO was performed as previously described (*28*). Poly-d-lysine–coated 384-well plates were manually seeded with 1.25 pmole of siRNA. Nontargeting siRNA and siRNAs targeting NIPBL, WAPL, and PDS5A/B were included as controls. Each siRNA was tested with four replicates. siRNA transfection was conducted as in the primary screen, except 2500 wild-type HCT116 cells were transfected per well. After 3 days, cells were fixed with 4% paraformaldehyde, 0.1% Tween-20 in PBS for 10 min at room temperature. The plates were rinsed with PBS and washed twice for 5 min each with PBS. Cells were then incubated with 70% ethanol, and the plates were sealed with foil plate covers, followed by incubation at 4°C for at least 20 hours. Next day, ethanol was aspirated, and PBS was added, followed by incubation for 10 min at room temperature. After rinsing once with PBS, cells were permeabilized with 0.5% Triton X-100 in PBS for 15 min, followed by incubation with 2× SSCT (0.3 M NaCl, 0.03 M sodium citrate, and 0.1% Tween-20) for 5 min at room temperature. Cells were then incubated with 2× SSCT/50% formamide (Thermo Fisher Scientific, no. BP227500), and the plates were sealed with foil plate covers. Predenaturation was performed by placing the plates on heat blocks at 91°C for 3 min, followed by incubation on heat blocks at 60°C for 20 min. The plates were centrifuged, and hybridization mixture was added to each well. The hybridization mixture contained 50% formamide, 10% dextran sulfate (Sigma-Aldrich, no. S4030), 4% polyvinylsulfonic acid (Sigma-Aldrich, no. 278424), 0.1% Tween-20, 2× SSC, and 2 pmole of each probe. After the centrifugation, the plates were sealed with foil covers and placed on heat blocks at 91°C for 20 min for denaturation. The plates were centrifuged and placed on heat block at 37°C for at least 20 hours. Next day, cells were rinsed twice with 2× SSCT at room temperature, washed once with 2× SSCT at 60°C for 5 min, and then washed again with 2× SSCT at room temperature for 5 min. Cells were stained with DAPI (1 μg/ml; Sigma-Aldrich, no. D5942) in 2× SSCT for 5 min, followed by a final wash with 2× SSC for 15 min. The plates were stored at 4°C until imaging.

For imaging, the plates were mounted with imaging buffer as described for the primary screen. DAPI and FISH images were acquired using a Molecular Devices ImageXpress Micro 4 high-content confocal microscope with a 0.45-μm pinhole and a 1.4 NA 60× water-immersion objective. Maximum projections of *z* stacks (six images, 0.5 μm spacing) were automatically generated in MetaXpress.

Images were analyzed using CellProfiler software (version 4.2.1), as described before with some modifications (*28*). Individual nuclei were segmented from DAPI images using two-class Otsu thresholding method. FISH spots were detected using the “robust background” thresholding method, and only spots located within nuclei were included in the analysis. Two dots on the same allele were paired by destructively iterating through all possible pairs and applying an empirical CC distance cutoff, as described previously (*28*). The coordinates of each dot were obtained using CellProfiler and the distances between paired dots were calculated.

### Co-IP

Approximately 10 million HCT116 cells were resuspended in buffer A [10 mM Hepes (pH 7.9), 1.5 mM MgCl2, 10 mM KCl, 340 mM sucrose, 10% glycerol, and protease inhibitor (Roche, no. 11836170001)] with Triton X-100 added to a final concentration of 0.1%. Cells were vortexed for 5 s and incubated for 5 min on ice. Nuclei were collected by centrifugation at 1300*g* for 4 min at 4°C. Nuclei were lysed in lysis buffer [10 mM Hepes (pH 7.9), 3 mM MgCl2, 5 mM KCl, 140 mM NaCl, 0.1 mM EDTA, 0.5% NP-40, protease inhibitor, and 1000 U of benzonase (Sigma-Aldrich, no. E1014)]. After incubation for 45 min at 4°C, the lysate was collected by centrifugation at 13,200 rpm for 20 min at 4°C. Antibodies (2 μg) were conjugated to 10 μl of protein G Dynabeads (Thermo Fisher Scientific, no. 10003D) for 1 hour at room temperature, then incubated with the lysate overnight at 4°C. Beads were washed four times with lysis buffer and proteins were eluted into sample buffer [NuPAGE LDS sample buffer (Invitrogen, no. NP0007) and NUPAGE reducing reagent (Invitrogen, no. NP0009)]. Samples were boiled for 5 min and analyzed by immunoblotting.

### Fractionation of chromatin-bound cohesin

Approximately 1 million HCT116 cells were washed once with cold PBS and resuspended in lysis buffer [10 mM Hepes (pH 7.9), 3 mM MgCl2, 5 mM KCl, 140 mM NaCl, 0.1 mM EDTA, 0.5% NP-40, and protease inhibitor]. After incubation for 20 min at 4°C, samples were centrifuged at 5000 rpm for 5 min at 4°C. The soluble fraction was discarded, and the pellet was resuspended in the same lysis buffer supplemented with 31.25 U of benzonase. Following the incubation for 45 min at 4°C, samples were centrifuged at 13,200 rpm for 20 min at 4°C. The cleared supernatant was mixed with NUPAGE LDS Sample Buffer and NUPAGE reducing reagent, then boiled for 5 min. For immunoblotting, nontargeting control samples were loaded in three independent wells, and the average band intensity was used to calculate fold change for each siRNA-targeted sample.

### Sample preparation to assess total protein levels

Approximately 1 million HCT116 cells were washed once with cold PBS and resuspended in lysis buffer [10 mM Hepes (pH 7.9), 3 mM MgCl2, 5 mM KCl, 140 mM NaCl, 0.1 mM EDTA, 0.5% NP-40, protease inhibitor, and 31.25 U of benzonase]. After incubation for 45 min at 4°C, samples were centrifuged at 13,200 rpm for 20 min at 4°C. The cleared supernatant was mixed with NUPAGE LDS Sample Buffer and NUPAGE reducing reagent, then boiled for 5 min. For immunoblotting, nontargeting control samples were loaded in three independent wells, and the average band intensity was used to calculate fold change for each siRNA-targeted sample.

### Immunoblotting

Lysates and IP samples were run on 4 to 12% bis-tris protein gels (Invitrogen, no. WG1403). Membranes were incubated with primary antibodies in 5% BSA in tris-buffered saline with 0.1% Tween-20 overnight at 4°C. Secondary antibodies used included anti-rabbit immunoglobulin G (IgG), horseradish peroxidase (HRP)–linked antibody (1:5000, Cell Signaling, no. 7074) for chemiluminescence imaging, and IRDye 800CW Donkey anti-Mouse IgG (1:10,000, LI-COR, no. 926-32212) and IRDye 680RD Donkey anti-rabbit IgG (1:10,000, LI-COR, no. 926-68073) for near-infrared fluorescence imaging. For HRP detection, SuperSignal West Pico (Invitrogen, no. 34080) or Femto (Invitrogen, no. 34095) substrates were used, and images were acquired using the ImageQuant LAS4000. For near-infrared fluorescence imaging, the LI-COR Odyssey Clx Imager was used. Antibody information can be found in table S3.

### Inhibitor treatment

Rotenone (Sigma-Aldrich, no. R8875) was used at 100 nM for 72 hours. TH287 (MedchemExpress, no. HY-16965) was used at 1 μM for 72 hours. PCAF-IN-1 (MedchemExpress, no. HY-147894), NSC694621 (MedchemExpress, no. HY-147288), and Garcinol (MedchemExpress, no. HY-107569) were used at 2, 10, and 5 μM for 72 hours, respectively. T2AA (MedchemExpress, no. HY-110111) was used at 40 μM for 24 hours.

### CRISPR KO in WAPL degron cells

HCT116:WAPL-AID cells were infected with lentivirus expressing sgRNAs targeting screening hits. Following 2 days of puromycin selection, cells were seeded on poly-d-lysine–coated chamber slides (Cellvis, no. C18SB-1.5H) and treated with 1 μM auxin for 3 days. Cells were then fixed and immunostained.

### RNA extraction and qPCR

RNA was extracted using the RNeasy Mini Kit (Qiagen, no. 74104) according to the manufacturer’s instructions. For cDNA synthesis, 1 μg of RNA was reverse transcribed using the iScript Reverse Transcription Supermix (Bio-rad, no. 1708841). Quantitative reverse transcriptase polymerase chain reaction was performed with SYBR Green Master Mix (Applied Biosystems, no. A46112) on a CFX Opus Real-Time PCR System (Bio-rad). β-Actin was used as an internal control for normalization. Primer sequences are listed in table S3.

### Flow cytometry

Cells were fixed in 1% paraformaldehyde in PBS for 10 min at room temperature and washed twice with fluorescence-activated cell sorting (FACS) buffer (5% BSA and 1 mM EDTA in PBS). Cells were permeabilized with cold methanol for 30 min on ice, followed by twice additional washes with FACS buffer. DNA was stained with 125 nM To-PRO-3 Iodide (642/661) (Invitrogen, no. T3605) in PBS for 1 hour at room temperature. Samples were analyzed on LSRFortessa flow cytometer (Becton Dickinson).

### Proliferation assay

HCT116:WAPL-AID cells (7 × 10^5^) were seeded in six-well plates. Next day, cells were treated with dimethyl sulfoxide (DMSO) and 1 μM auxin in fresh media. Every day, cells were trypsinized and their number was counted by Countess 2 (Invitrogen) with triplicates. The number of cells was divided by six-well surface area (9.6 cm^2^).

### Antibodies

The antibodies used in this study are listed in table S3.

### siRNA

Catalog number of siRNAs from Dharmacon used in primary and secondary screens, de-pooled siRNA validation, DNA FISH, CTCF clustering assays, and other experiments are provided in tables S1 to S3.
