# Supplementary material for: A genetic screen for modifiers of cohesin clustering identifies regulators of genome folding
Source: Sci Adv. 2026 Jan 30;12(5):eadx5130. doi: 10.1126/sciadv.adx5130 (PMC12857682; doi:10.1126/sciadv.adx5130)
Supplement: Supplementary file 1 — Figs. S1 to S11 Legends for tables S1 to S3 [file sciadv.adx5130_sm.pdf]

Science Advances
AAAS

Supplementary Materials for

A genetic screen for modifiers of cohesin clustering identifies regulators of genome folding

Wonho Kim *et al.*

Corresponding author: Wonho Kim, wonhok@unc.edu; Rajan Jain, jainr@pennmedicine.upenn.edu

Sci. Adv. **12**, eadx5130 (2026)
DOI: 10.1126/sciadv.adx5130

The PDF file includes:

Figs. S1 to S11
Legends for tables S1 to S3

Other Supplementary Material for this manuscript includes the following:

Tables S1 to S3

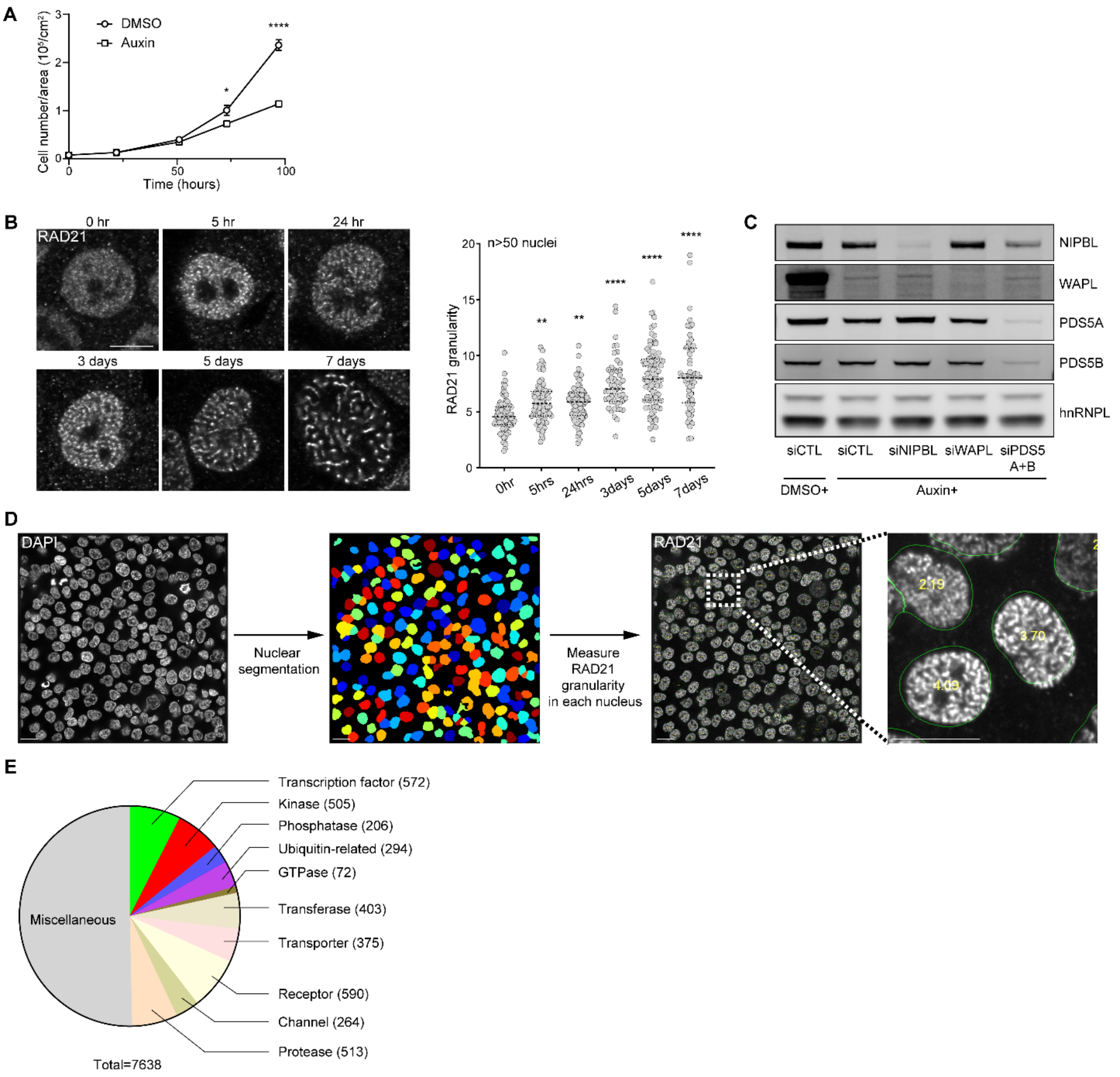

Fig. S1. Characterization of HCT116:WAPL-AID cells upon WAPL depletion.
(A) Cell proliferation assay of HCT116:WAPL-AID cells treated with DMSO or auxin. Cells were treated with 1 μM auxin for 97 hours, and cell numbers were quantified at the indicated time points. *, **** indicate $p<0.05$, $p<0.0001$, respectively, as determined by unpaired t test at each time point. n = 3. Error bars denote mean ± SD. (B) RAD21 immunostaining of HCT116:WAPL-AID cells treated with auxin over a time course. Cells were treated with 1 μM auxin for the indicated durations. Representative images of RAD21 immunostaining are shown on the left. Scale bar, 10 μm. Quantification of RAD21 granularity is shown on the right. Data points represent individual nuclei, with the median (thick dotted line) and interquartile range (thin dotted lines) indicated. **, **** indicate $p<0.01$, $p<0.0001$, respectively, as determined by ANOVA followed by Dunnett's test to compare conditions to 0 hr time point. n > 50. (C) Immunoblots of NIPBL, WAPL, PDS5A, PDS5B and hnRNPL from HCT116:WAPL-AID cells treated with auxin and transfected with non-

targeting (CTL) siRNA or siRNAs targeting NIPBL, WAPL and PDS5A/B. hnRNPL was used as a loading control. (D) Image analysis pipeline used in screening. Nuclei were segmented from DAPI images, and RAD21 granularity was measured per nucleus. Scale bar, 10 µm. (E) Gene ontology analysis of the screened library.

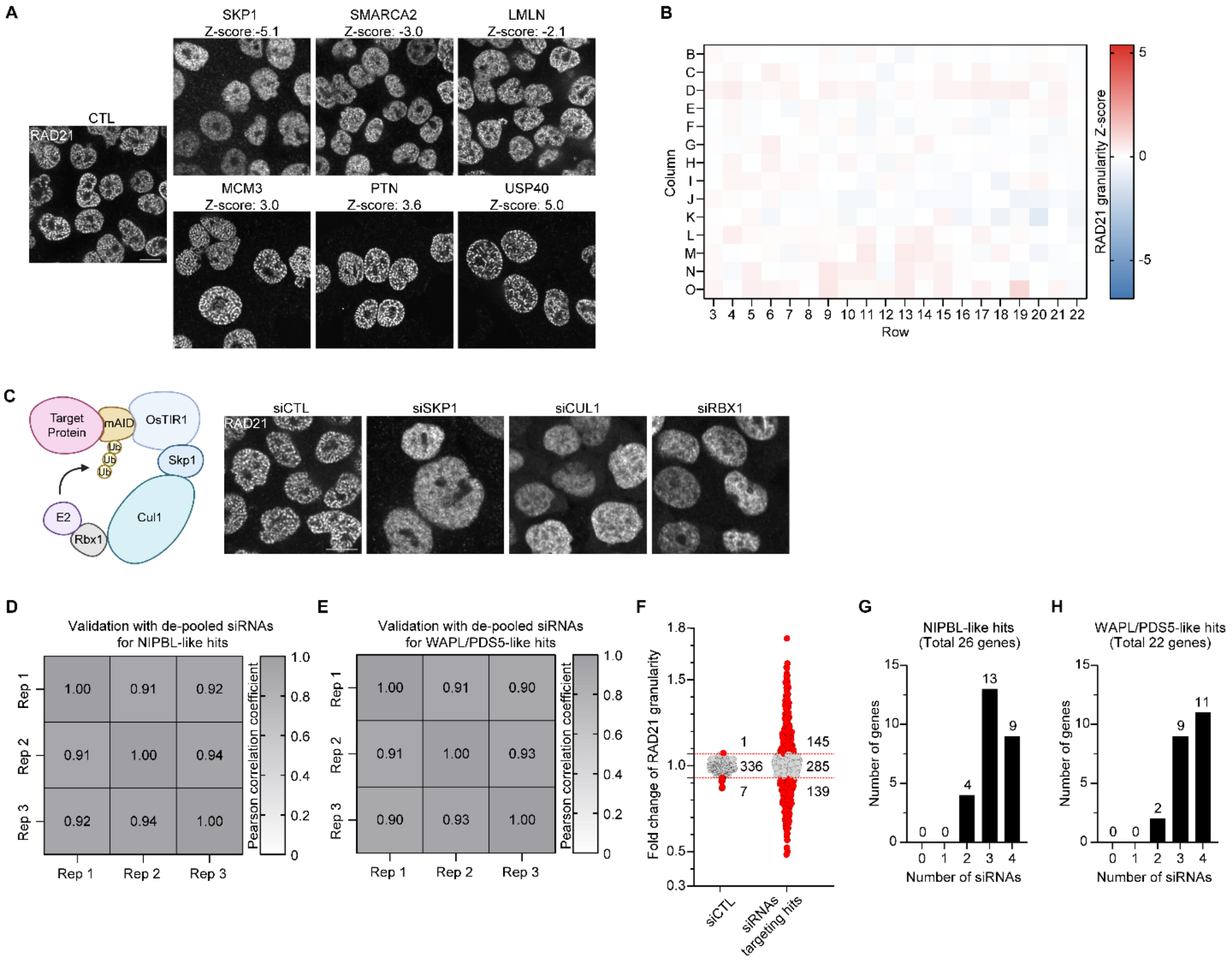

Fig. S2. Assessment of primary screen and identification of NIPBL- and WAPL/PDS5-like hits in primary screen.

(A) Representative RAD21 immunostaining images from the pooled siRNA primary screen, showing a range of z-scores. The gene name and z-score are indicated. Scale bar, 10 µm. (B) Heatmap showing average RAD21 granularity z-score across all 384-well plates in the pooled siRNA primary screen. Color intensity indicates the magnitude of z-score, ranging from the minimum (–6.84) to the maximum (5.37) observed in the primary screen. (C) RAD21 immunostaining of HCT116:WAPL-AID cells treated auxin and transfected with siRNAs targeting endogenous components of the auxin degron machinery or non-targeting (CTL) siRNA. The schematic on the left illustrates the auxin degron machinery. Created in BioRender. Kim, W. (2025) https://BioRender.com/asgb9za. Representative images were obtained from the pooled siRNA primary screen. Scale bar, 10 µm. (D,E) Heatmaps showing Pearson correlation between three replicates for de-pooled siRNA validation of NIPBL-like hits (D) and WAPL/PDS5-like hits (E). (F) Distribution plot of non-targeting siRNA replicates and siRNAs targeting screening hits. The fold change for each siCTL replicate and each siRNA targeting a screening hit was calculated relative to the median of all siCTL replicates. Red dotted lines indicate ±7% change, with data points outside this range highlighted as red dots. The number of data points in each category is indicated. (G,H) Numbers of genes with 0-4 individual siRNAs showing decreased RAD21

granularity for NIPBL-like hits (G) and increased RAD21 granularity for WAPL/PDS5-like hits (H).

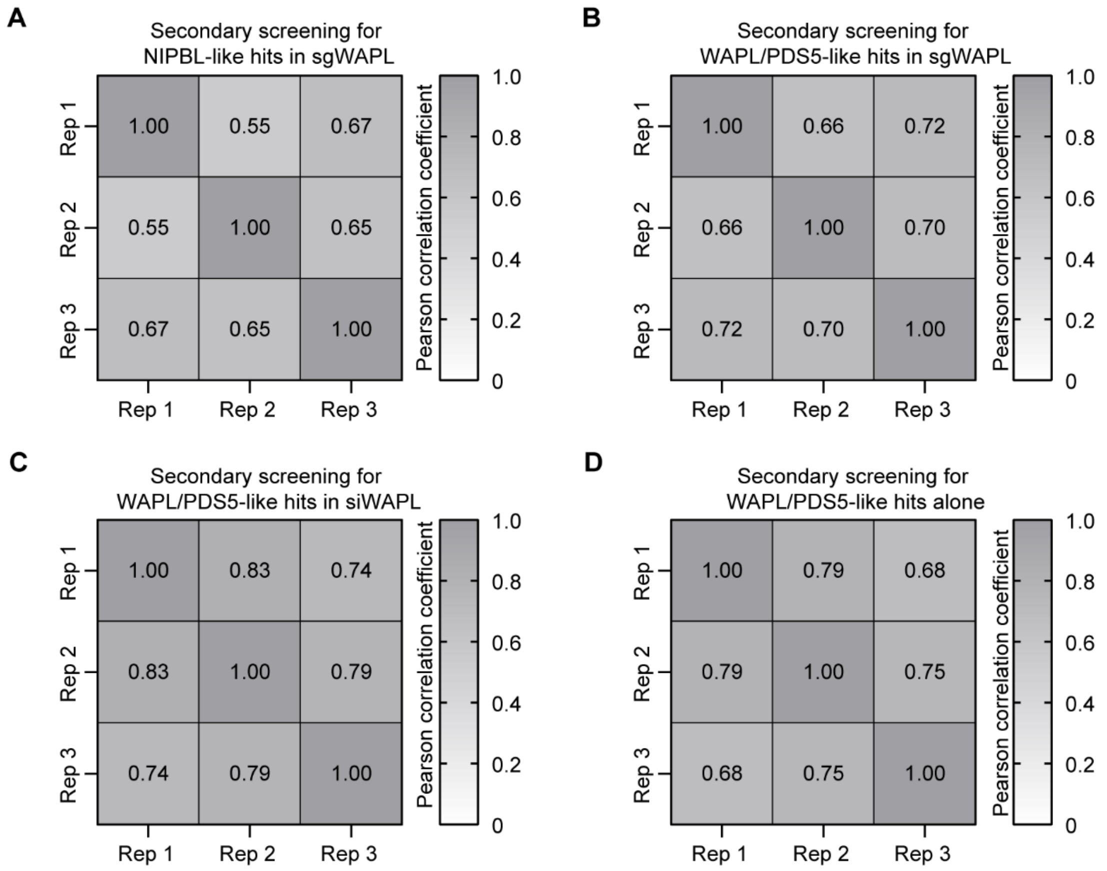

Fig. S3. Correlation between replicates in secondary screens.
(A) Heatmap showing Pearson correlation between three replicates for NIPBL-like hits in secondary screen using CRISPR WAPL KO. (B-D) Heatmaps showing Pearson correlation between three replicates for WAPL/PDS5-like hits in secondary screens using CRISPR WAPL KO (B), siRNA targeting WAPL (C) and siRNA targeting hit alone without WAPL depletion (D). Related to Table S2.

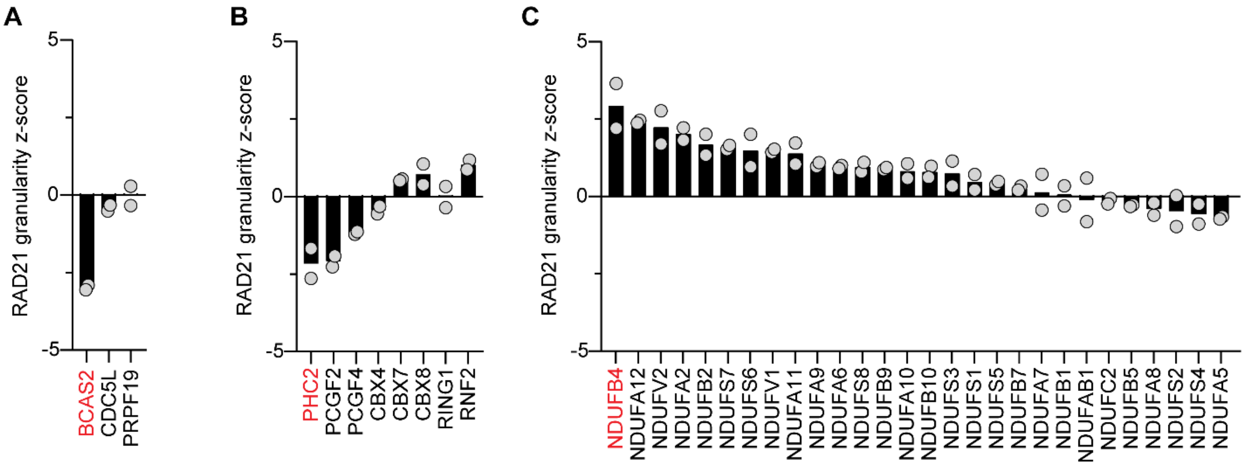

Fig. S4. Analysis of complexes containing screening hits.

(A-C) Complexes that include screening hits were analyzed, and the z-scores of their components in the pooled siRNA primary screen are shown. BCAS2-CDC5L-PRPF19 complex (A), cPRC1 (B), and mitochondrial complex I (C) are plotted. Data from two replicates of the pooled siRNA primary screen are shown. Screening hits are highlighted in red.

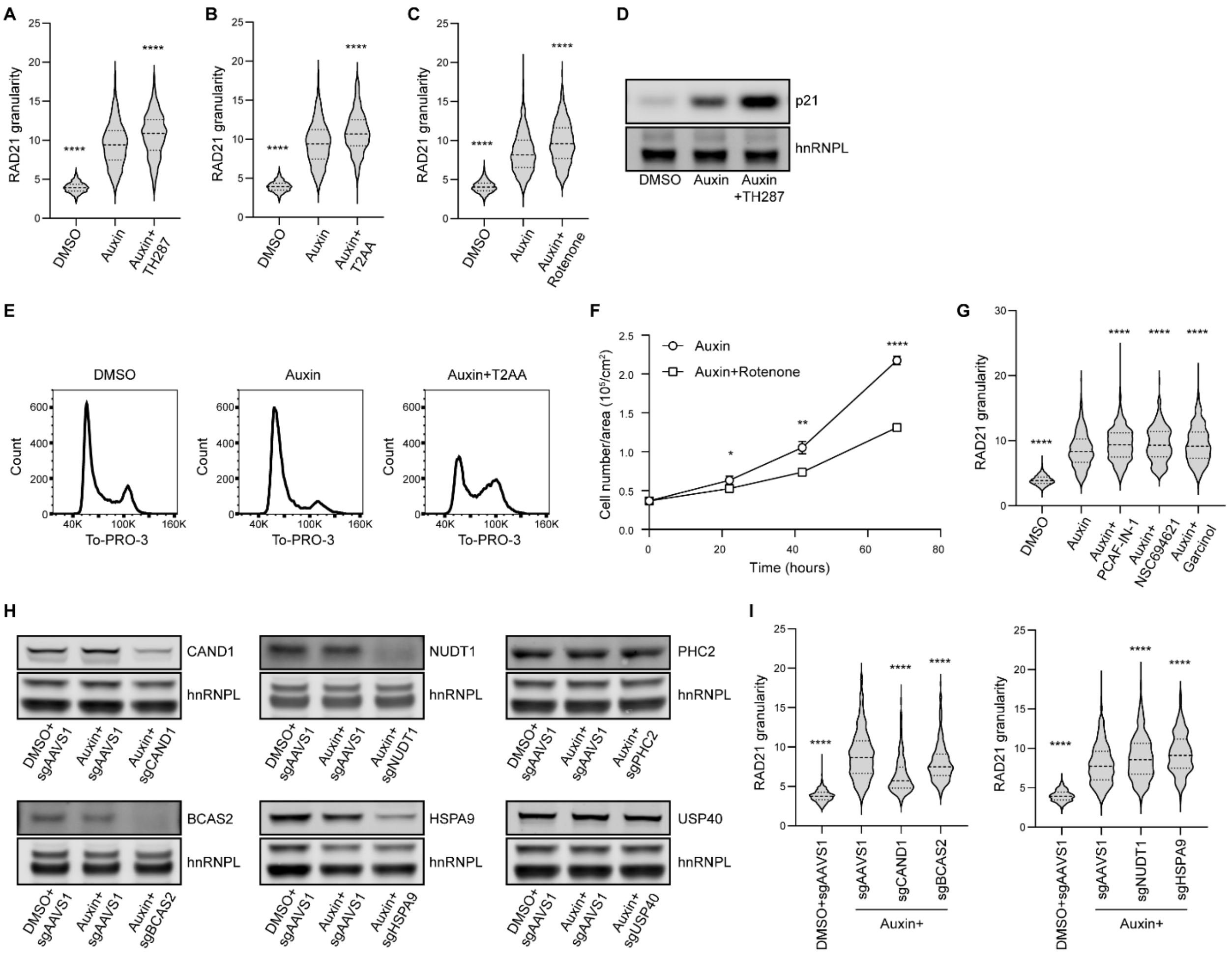

Fig. S5. Validation of candidate regulators by pharmacological and genetic approaches. (A-C) Quantification of RAD21 granularity in HCT116:WAPL-AID cells treated with DMSO or auxin (1 µM, 72 hours) together with small-molecule inhibitors: TH287 (1 µM, 72 hours; NUDT1 inhibitor; A), T2AA (40 µM, 24 hours; PCNA inhibitor; B) and rotenone (100 nM, 72 hours; mitochondrial complex I inhibitor; C). (D) Immunoblots of p21 from HCT116:WAPL-AID cells treated with auxin and TH287. p21 accumulation confirms NUDT1 inhibition by TH287, as reported previously (36). hnRNPL was used as a loading control. (E) Flow cytometry analysis of HCT116:WAPL-AID cells treated with auxin and T2AA. DNA was stained with To-PRO-3 iodide. 20000 cells plotted. PCNA inhibition by T2AA led to accumulation of S-phase cells, as reported previously (37). (F) Cell proliferation assay of HCT116:WAPL-AID cells treated with 1 µM auxin with or without 100 nM rotenone for 68 hours. Cell numbers were quantified at the indicated time points. *, **, **** indicate $p<0.05$, $p<0.01$, $p<0.0001$, respectively, as determined by unpaired t test at each time point. n = 3. Error bars denote mean ± SD. Cell proliferation was reduced, as reported previously (38). (G) Quantification of RAD21 granularity in HCT116:WAPL-AID cells treated with auxin and KAT2B inhibitors (2 µM PCAF-IN-1, 10 µM NSC694621 and 5 µM Garcinol, 72 hours). (H) Immunoblots of CAND1, BCAS2, NUDT1, HSPA9, PHC2 and USP40 in sgRNA-expressing cells treated with auxin (1 µM, 72 hours). hnRNPL was used as a loading control. (I) Quantification of RAD21 granularity in HCT116:WAPL-AID cells expressing sgRNAs targeting CAND1, BCAS2, NUDT1, or HSPA9 treated with auxin (1 µM, 72 hours). (A-

C,G,I) **** indicates $p<0.0001$, as determined by ANOVA followed by Dunnett's test to compare conditions to auxin treatment alone (A-C, G) or control sgRNA expression with auxin treatment (I).

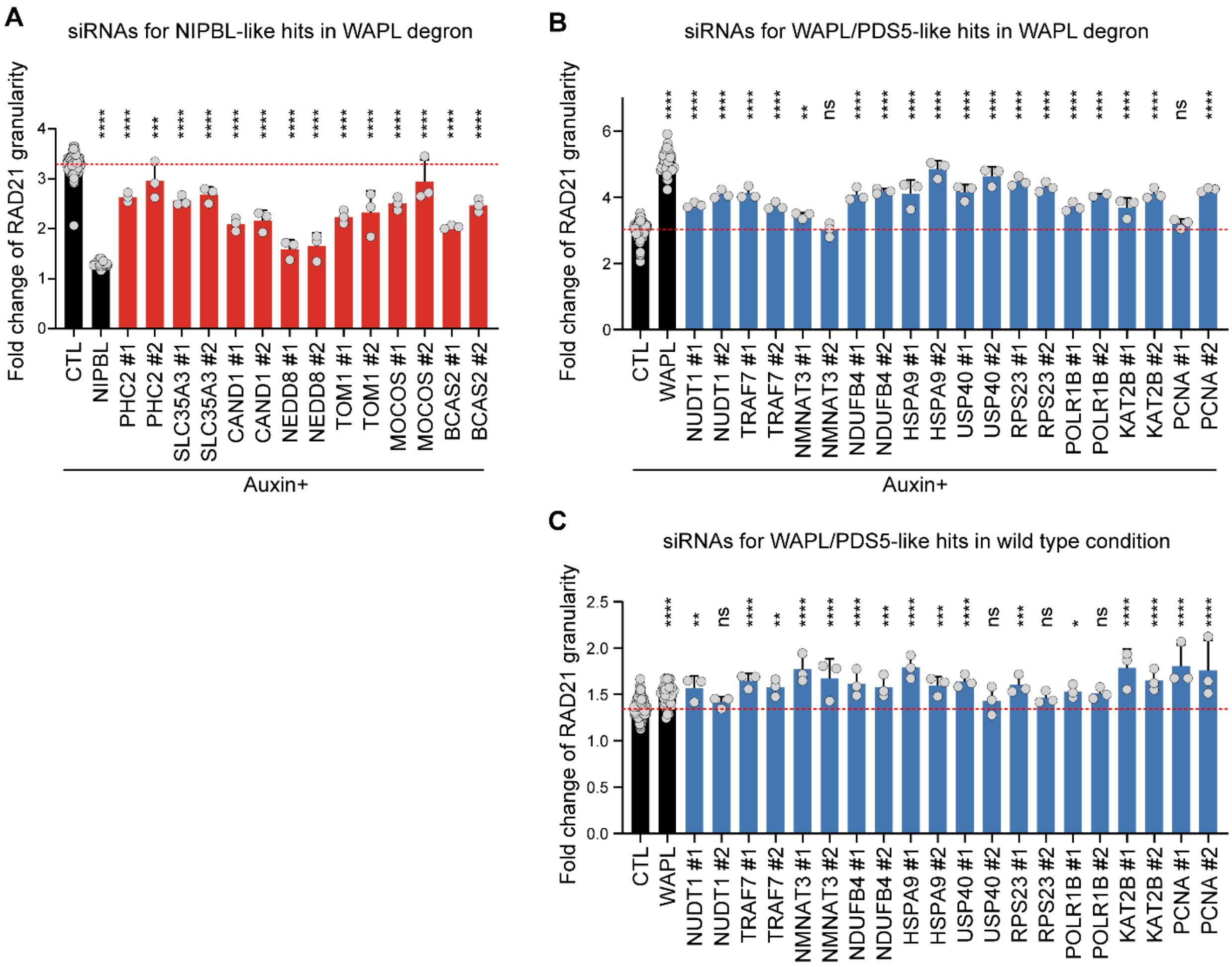

Fig. S6. Cohesin clustering upon knockdown of NIPBL- and WAPL/PDS5-like hits.
(A,B) Quantification of RAD21 granularity in HCT116:WAPL-AID cells treated with auxin and transfected with non-targeting (CTL) siRNA or siRNAs targeting NIPBL-like hits (A) and WAPL/PDS5-like hits (B). The mean of auxin treatment and siCTL is indicated by red dotted lines. (C) Quantification of RAD21 granularity in HCT116 cells transfected with non-targeting siRNA or siRNAs targeting WAPL/PDS5-like hits without WAPL depletion. The mean of siCTL is indicated by red dotted line. (A-C) Two siRNAs were tested per hit. *, **, ***, **** indicate $p<0.05$, $p<0.01$, $p<0.001$, $p<0.0001$, respectively; ns indicates non-significant, as determined by ANOVA followed by Dunnett's test to compare conditions to non-targeting siRNA. $n > 3$. Error bars denote mean + SD.

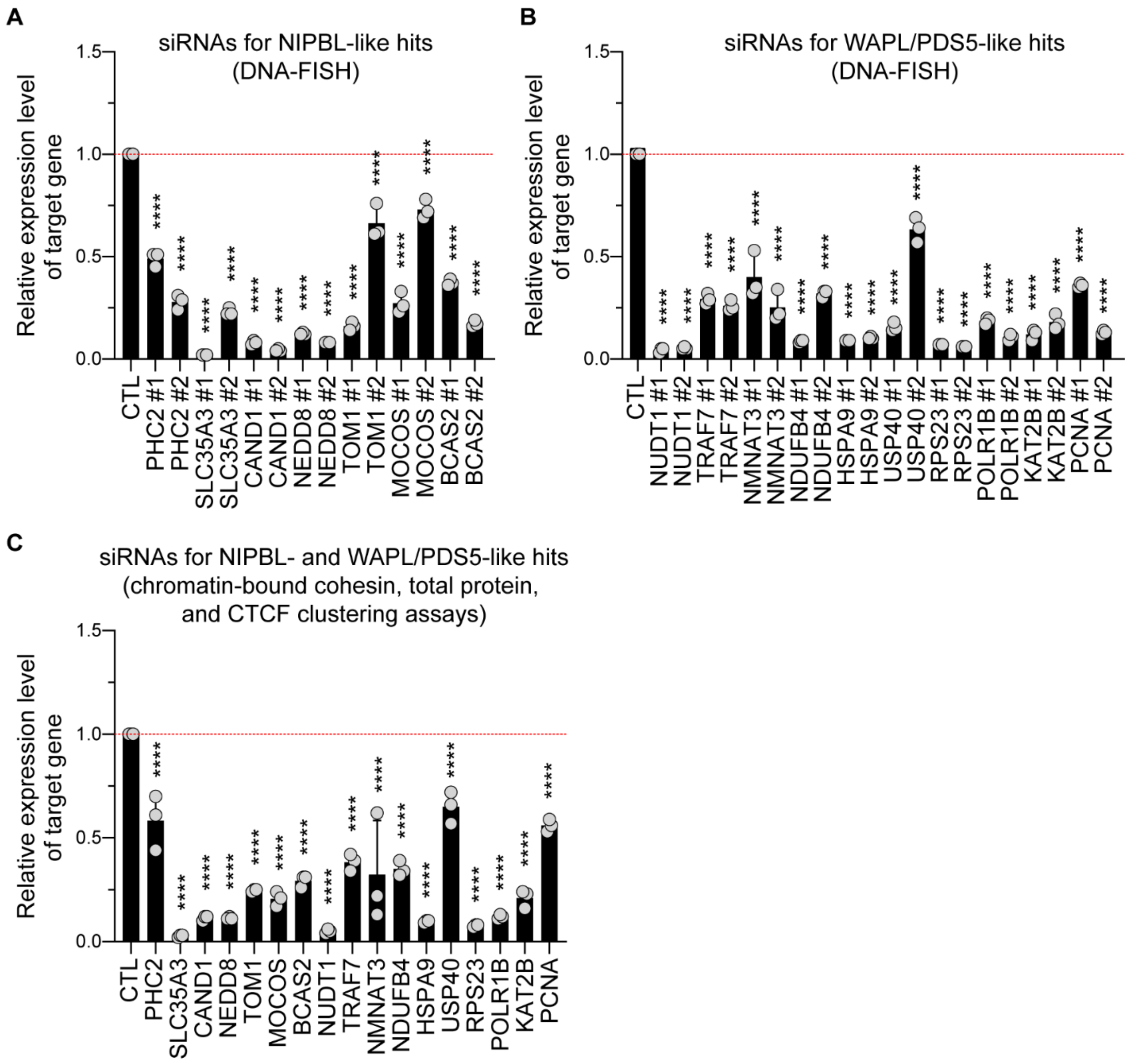

Fig. S7. Confirmation of knockdown of NIPBL- and WAPL/PDS5-like hits.
(A,B) Relative expression levels of NIPBL-like hits (A) and WAPL/PDS5-like hits (B) in DNA FISH experiments, normalized to non-targeting (CTL) siRNA control. Two siRNAs were tested per hit. (C) Relative expression levels of NIPBL- and WAPL/PDS5-like hits in chromatin-bound cohesin, total protein and CTCF clustering assays, normalized to siCTL. (A-C) n = 3 (technical replicates). **** indicates $p<0.0001$, as determined by ANOVA followed by Dunnett's test to compare conditions to non-targeting siRNA control. Error bars denote mean + SD.

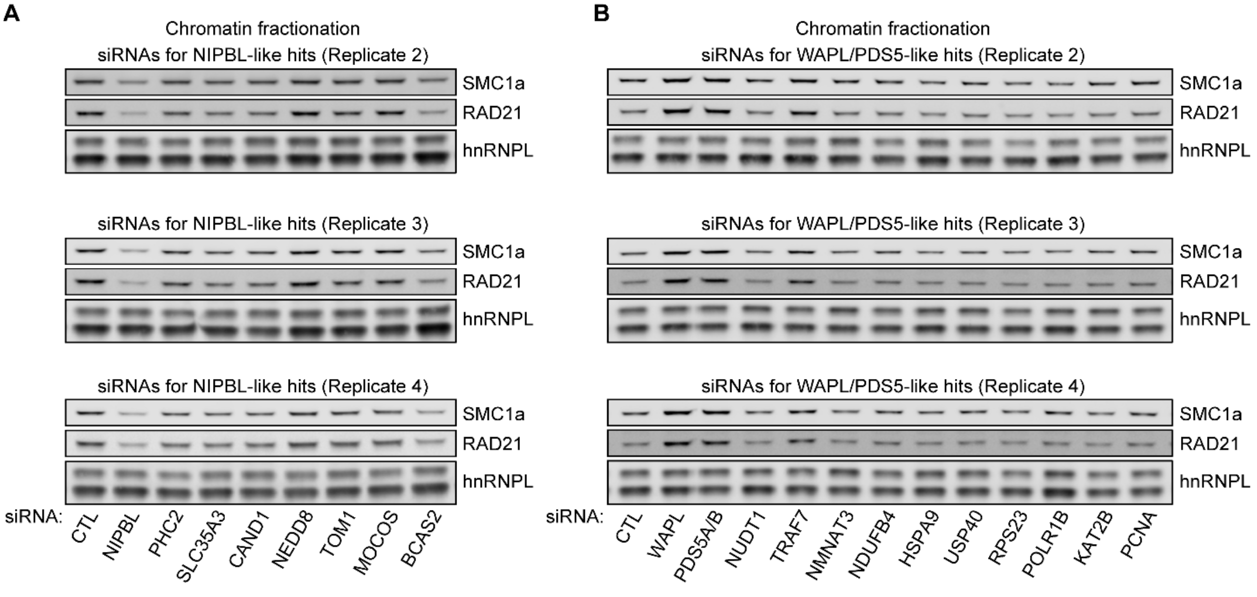

Fig. S8. Examination of chromatin-bound cohesin upon knockdown of screening hits. (A,B) Immunoblots of SMC1a and RAD21 from HCT116 cells transfected with siRNAs targeting NIPBL-like hits (A) or WAPL/PDS5-like hits (B). Chromatin fractions were analyzed. One biological replicate is shown in Fig. 6, and three additional biological replicates were shown here. hnRNPL was used as a loading control.

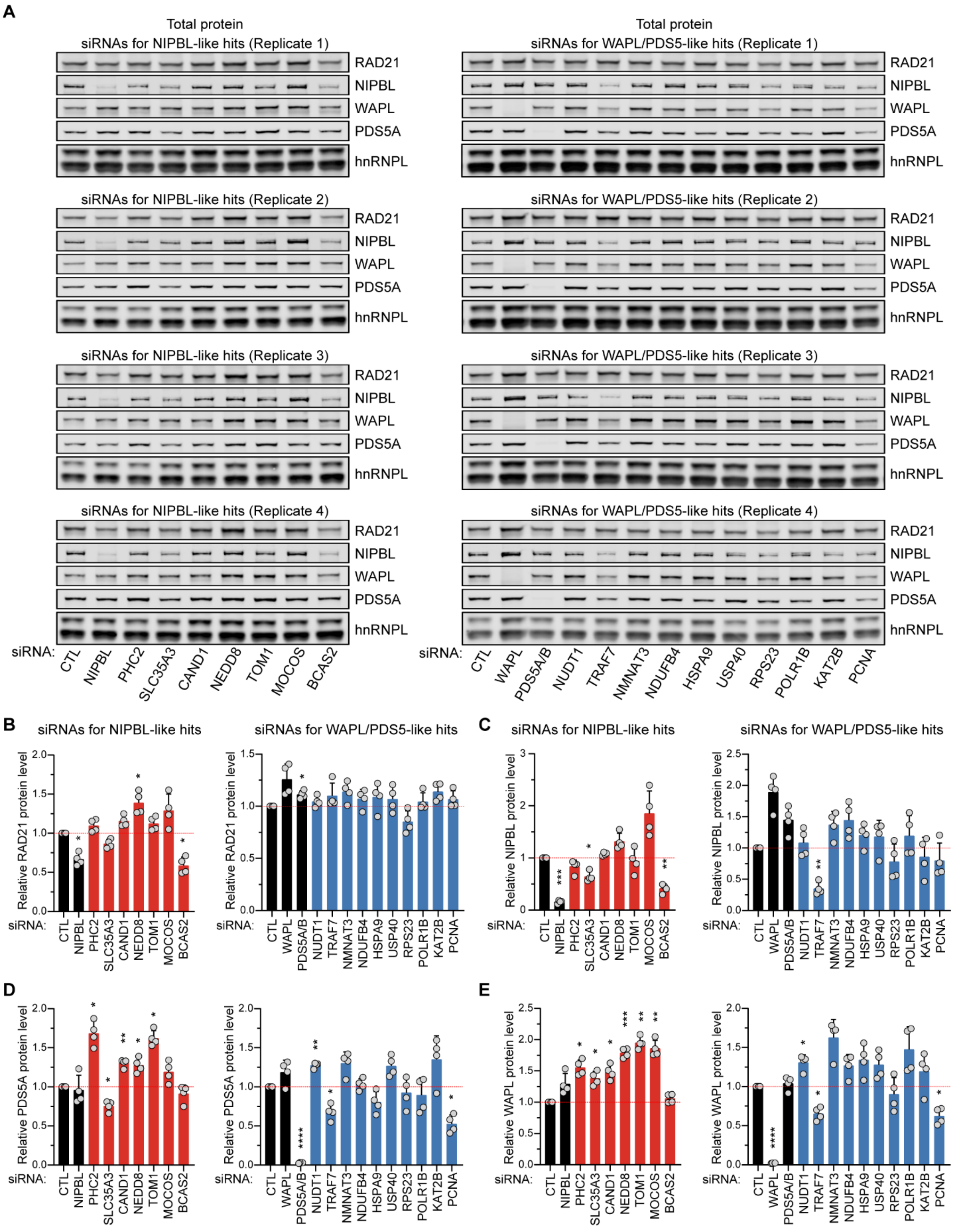

Fig. S9. Total protein levels of cohesin and cohesin-associated factors upon knockdown of screening hits.

(A) Immunoblots of RAD21, NIPBL, WAPL, and PDS5A from HCT116 cells transfected with non-targeting (CTL) siRNA or siRNAs targeting NIPBL-like hits (left) or WAPL/PDS5-like hits (right). Whole-cell lysates were analyzed. hnRNPL was used as a loading control. Four biological replicates are shown. (B-E) Quantification of RAD21 (B), NIPBL (C), PDS5A (D), and WAPL (E). Band intensities were normalized to hnRNPL. *, **, ***, **** indicate $p<0.05$, $p<0.01$, $p<0.001$, $p<0.0001$, respectively, as determined by ANOVA followed by Dunnett's test to compare to siCTL. n = 4. Error bars denote mean + SD.

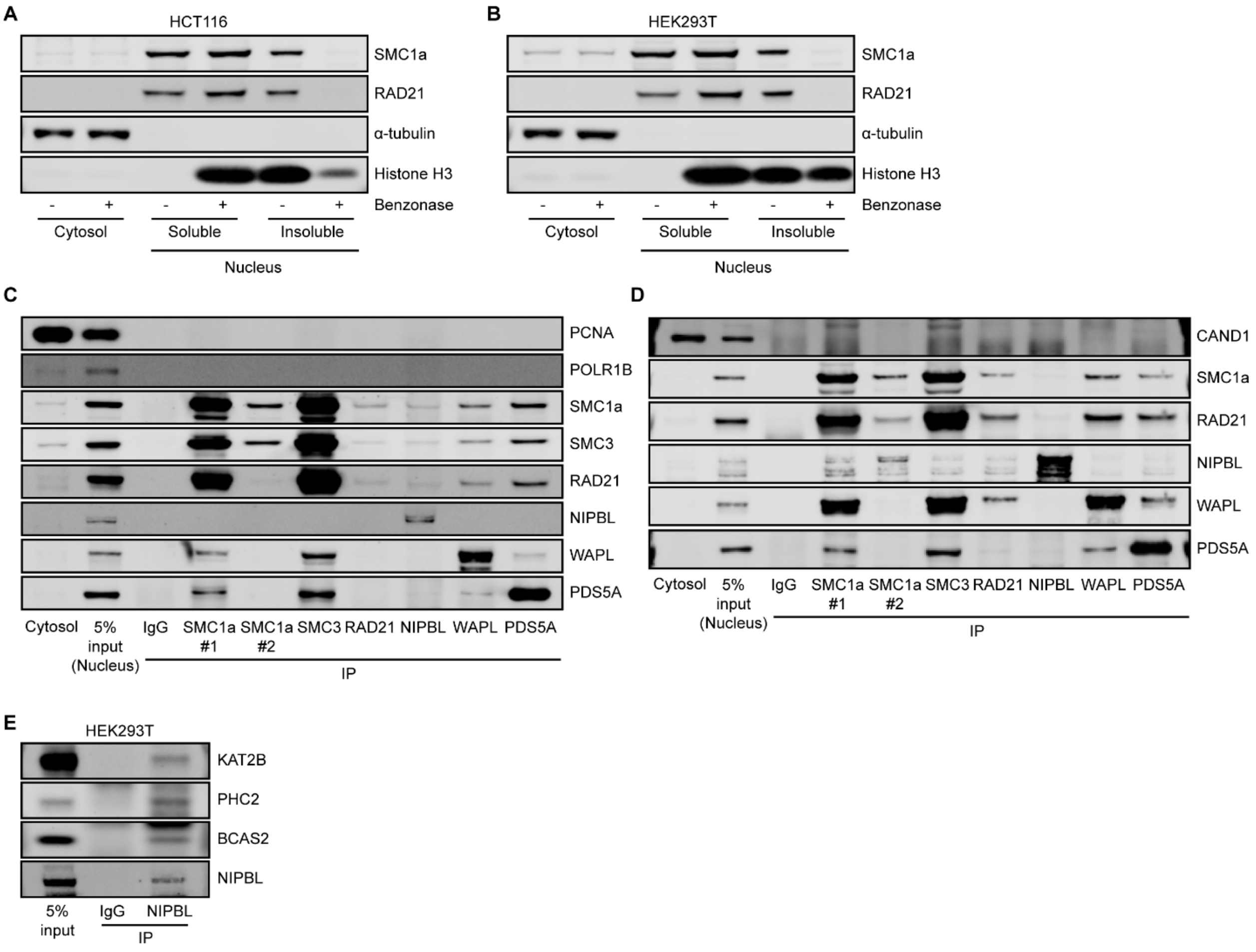

Fig. S10. Test interaction of candidate proteins with cohesin and cohesin-associated factors. (A,B) Cytosolic, soluble nuclear and insoluble nuclear fractions with or without benzonase treatment were analyzed by immunoblotting for SMC1a, RAD21, α-tubulin (cytosolic marker) and Histone H3 in HCT116 (A) and HEK293T (B) cells. (C,D) Immunoprecipitation of cohesin and cohesin-associated factors from HCT116 cells, followed by immunoblots analysis using the indicated antibodies to detect interactions with PCNA, POLR1B, and CAND1. Two antibodies for SMC1a were used for immunoprecipitation. Blots for cohesin, NIPBL, WAPL and PDS5A in (D) were reused from Fig. 7E. (E) Immunoprecipitation of NIPBL, followed by immunoblot analysis using the indicated antibodies in HEK293T cells.

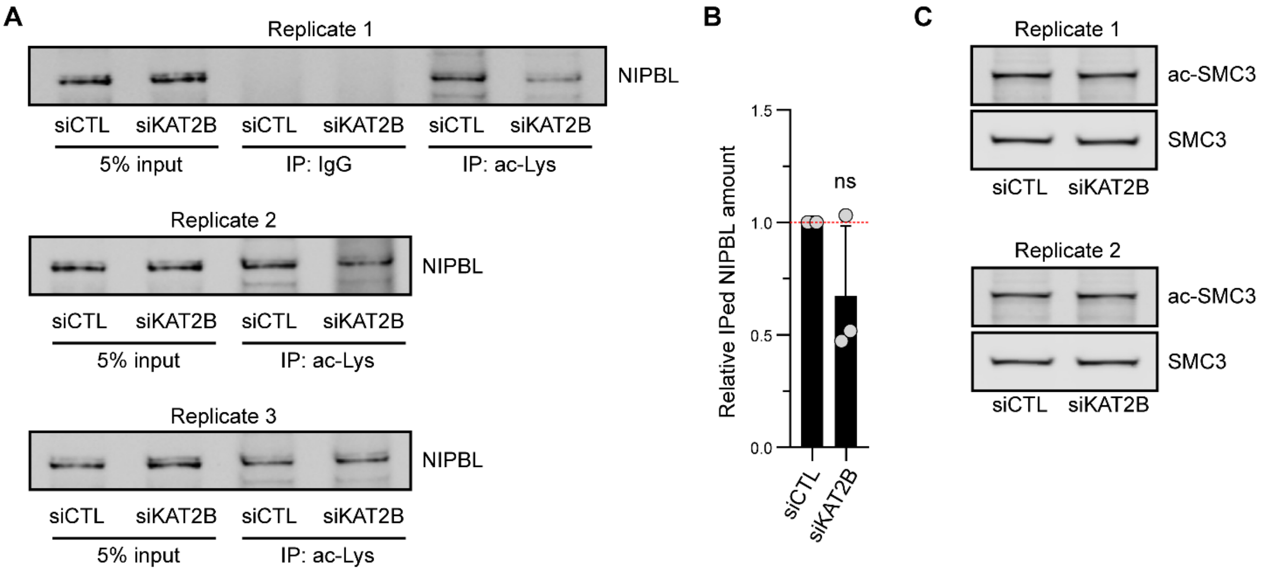

Fig. S11. NIPBL and SMC3 acetylation upon KAT2B knockdown.
(A) Immunoprecipitation of acetylated proteins, followed by immunoblot analysis using NIPBL antibody in HCT116 cells transfected with KAT2B siRNA. Three biological replicates are shown. (B) Quantification of immunoprecipitated NIPBL from (A). No significant difference was detected by paired t test compared to non-targeting (CTL) siRNA. n = 3. Error bars denote mean + SD. (C) Immunoblots of acetylated SMC3 and total SMC3 from HCT116 cells transfected with KAT2B siRNA. Two biological replicates are shown.

Supplementary Table 1.
Primary screen data, related to Figure 1. This table contains data for all genes tested with pooled siRNA, including z-scores of RAD21 granularity and the number of nuclei analyzed for each replicate. Data from de-pooled siRNAs for 80 suppressors and 38 enhancers are also provided, including RAD21 granularity, fold change in RAD21 granularity compared to non-targeting siRNA, and the number of nuclei analyzed for each replicate. Primary screen hits comprise 26 NIPBL-like and 22 WAPL/PDS5-like genes.

Supplementary Table 2.
Secondary screen data, related to Figure 2 and 3. This table contains data for 26 NIPBL-like and 22 WAPL/PDS5-like hits prioritized from the primary screen. They include RAD21 granularity, fold change in RAD21 granularity compared to non-targeting siRNA, and the number of nuclei analyzed for each replicate. Data includes knockdown of screening hits in WAPL KO cells (NIPBL-like hits) and in conditions with WAPL KO, siWAPL or no WAPL depletion (WAPL/PDS5-like hits). Final hits comprise 7 NIPBL-like, 9 WAPL-like, and 1 PDS5-like gene.

Supplementary Table 3.
DNA FISH probe design coordinates (hg38), and lists of siRNAs, antibodies and primers used in this study.
